# A New Class of Carbon Nanostructures for High‐Performance Electro‐Magnetic and ‐Chemical Barriers

**DOI:** 10.1002/advs.202102718

**Published:** 2021-09-30

**Authors:** Jae Hui Park, Yun Ji Oh, Dong Yoon Park, Joonsik Lee, Jae Seo Park, Chong Rae Park, Jae Ho Kim, Taehoon Kim, Seung Jae Yang

**Affiliations:** ^1^ Advanced Nanohybrids Laboratory Department of Chemistry and Chemical Engineering Education and Research Center for Smart Energy and Materials Inha University Incheon 22212 Republic of Korea; ^2^ Carbon Nanomaterials Design Laboratory Research Institute of Advanced Materials Department of Materials Science and Engineering Seoul National University Seoul 08826 Republic of Korea; ^3^ Composites Research Division Korea Institute of Materials Science (KIMS) Changwon 51508 Republic of Korea

**Keywords:** carbon nanostructures, electrocatalytic effect, electromagnetic wave absorption, functional nanomaterials, lithium–sulfur batteries

## Abstract

It is of importance to explore a new carbon nanomaterial possessing vital functions to fulfill the high standards for practical achievement of the electromagnetic (EM) barrier for blocking EM waves and the electrochemical (EC) barrier as a functional separator for EC energy storage. Herein, facile synthesis of a new class of carbon nanostructures, which consist of interconnected N‐doped graphitic carbon nanocubes partially embedded by nickel nanoparticles, is described. The hollow interior of graphitic nanocube induces internal reflection of EM waves and confines active materials of EC energy storage. Nitrogen functionalities implanted in graphitic structure enhance electrical conductivity as well as improve chemical interaction with active materials. Furthermore, nickel nanoparticles in graphitic nanocube function as an EM wave‐absorbing material and an electrocatalyst for EC energy storage. Through comprehensive assessments, remarkable performances originating from distinctive nanostructures give new insights into structural design for the carbon nanostructure‐based high‐performance EM and EC barriers.

## Graphical abstract

A novel carbon nanostructure is demonstrated as a promising key material for electromagnetic (EM) and electrochemical (EC) applications. Distinctive structural features originating from interconnected N‐doped graphitic nanocubes embedding nickel nanoparticles impart crucial functions desirable for the EM and EC barriers. Outstanding performances of the resulting carbon nanostructure are substantiated through comprehensive investigation and assessments for the EM and EC barriers.

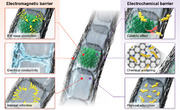

## 1 Introduction

Since the monumental discovery of fullerenes, carbon nanomaterials have attracted significant attention in the field of materials research beyond the traditional carbon forms.^[^ ^1^, ^2^, ^3^ ^]^ Research on carbon nanomaterials have resulted in the development of different allotropes with distinct dimensions, from 0D to 3D.^[^ ^4^ ^]^ The dimension‐derived structural features coupled with the superior physicochemical properties of the carbon nanomaterials impart it with great potential for use as a core material for next‐generation energy and environmental technologies.^[^ ^5^, ^6^, ^7^ ^]^ Among various technologies, their notable characteristics appear to be magnified both in electromagnetic (EM) and electrochemical (EC) applications.^[^ ^8^, ^9^, ^10^ ^]^

With the ever‐growing use of electronics, it is inevitable to be exposed to EM waves arising from electronics; therefore, EM interference has become a critical issue in modern times.^[^ ^11^ ^]^ In particular, EM waves can impair the operation of battery management system which prevents malfunction of battery in electric vehicles.^[^ ^12^ ^]^ In addition, this issue can be exacerbated when developing military applications, such as stealth technology in which radar absorbing materials are utilized to cancel reflections of a radar signal. Carbon materials have gained considerable attention in the field of EM wave absorption because of their advantageous features, including high electrical conductivity and light weight.^[^ ^13^ ^]^ The absorption performance of carbon materials, however, is not sufficient to fulfill the high standards for practical implementation because carbon materials, in general, can be exploited only with high permittivity, which prevents impedance matching and reflects the EM waves.^[^ ^14^, ^15^ ^]^ Therefore, the development of a new class of carbon materials, being endowed with magnetic property and other additional relaxation process, is highly desirable.

Meanwhile, the demand for sustainable energy sources for electric vehicles and grid energy storage has generated the need for advanced EC energy storage systems.^[^ ^16^, ^17^ ^]^ Among them, lithium–sulfur (Li–S) batteries are promising candidates due to their high energy density (2500 Wh kg^–1^) and the natural abundance of sulfur ensuring low cost.^[^ ^18^, ^19^, ^20^ ^]^ However, Li–S batteries still face severe issues related to deteriorating EC performance due to its complicated reaction mechanism.^[^ ^21^, ^22^ ^]^ Functional separator is an emerging concept as an EC barrier, performing the vital roles of preventing the passage of active materials through the separator and promoting the electrochemical reaction. Numerous carbon nanomaterials have been studied and applied in the functional separator as an upper current collector to provide an electrical pathway and trap lithium polysulfides (LiPSs).^[^ ^23^ ^]^ Despite great progress, crucial functions have to be equipped with carbon materials, such as chemical anchoring and catalytic effect, to innovate a carbon‐based functional separator.^[^ ^24^ ^]^ Thus, exploring a new class of carbon nanostructures possessing the desired functions is of great concern in both research fields to overcome the limitations of the existing carbon nanostructures.

Herein, we demonstrate a new class of 1D carbon nanostructures consisting of interconnected graphitic nanocubes as a promising candidate for high‐performance EM and EC barriers. Melamine, a nongraphitizable organic compound, can be reorganized into an unprecedented graphitic nanostructure through a coordination reaction with nickel ions, followed by a phase transition step and carbonization.^[^ ^25^ ^]^ The basic unit of the nanostructure is a hollow graphitic nanocube, which provides meso‐ and macroporous space to induce internal reflection of EM waves and confine the active materials for Li–S batteries. Nitrogen implanted in a highly crystalline graphitic surface endows additional surface functionalities to the resulting materials. Furthermore, Ni nanoparticles, which act as a catalyst to promote low‐temperature graphitization and partly remain in the resulting graphitic nanocubes, perform unexpected functions of wave absorption and catalytic effect for the EM and EC barriers, respectively. We validate the synthesis based on an unusual melamine‐nickel coordination reaction can realize multifarious features in one material, thereby producing a promising candidate to satisfy the criteria for both high‐performance EM and EC barriers.

## 2 Results and Discussion

### 2.1 Synthetic Procedure and Characterization of Precursor Preparation

**Figure 1:**
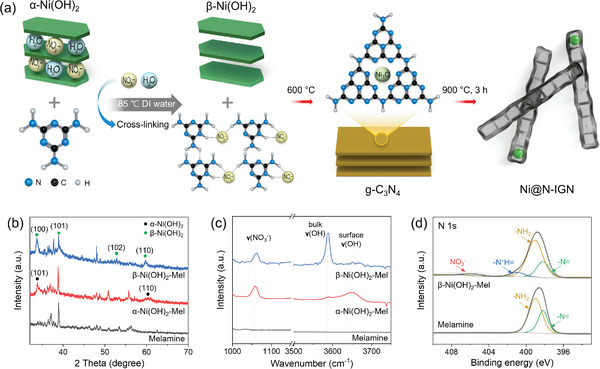
a) Schematics describing the synthetic process of Ni@N‐IGN. Enlarged b) XRD patterns and c) Raman spectra of melamine, *α*‐Ni(OH)_2_‐Mel, and *β*‐Ni(OH)_2_‐Mel. d) XPS N 1s spectra of melamine and *β*‐Ni(OH)_2_‐Mel.

As shown in **Figure** **1**a, the synthesis was proceeded by precursor preparation and subsequent heat treatment. The precursor was prepared through the wet chemical procedure and aging step of melamine and nickel nitrate. After the addition of NaOH to the solution of melamine and nickel nitrate, homogeneous coordinative compounds were precipitated, which were formed by interaction between nickel and amino groups (—NH_2_).^[^ ^26^, ^27^ ^]^ During the aging step in a convection oven at 85 °C, NO_3_^–^ ions and H_2_O molecules inside the *α*‐Ni(OH)_2_ frame were removed, which resulted in phase transition of *α*‐Ni(OH)_2_ to *β*‐Ni(OH)_2_,^[^ ^28^ ^]^ while melamine became protonated and bound to the NO_3_^–^ ions.^[^ ^29^, ^30^ ^]^ In the subsequent heat treatment under N_2_ atmosphere, *β*‐Ni(OH)_2_‐Mel was converted to graphitic carbon nitride (g‐C_3_N_4_) and nickel carbide at 600 °C. Nickel carbide was reduced to nickel nanoparticles at higher temperatures, while Ni‐catalyzed graphitization occurred around Ni, thereby completing the final structure in which the graphitic nanocubes were interconnected. The final carbon nanostructure composed of interconnected N‐doped graphitic nanocube with Ni nanoparticles is denoted as Ni@N‐IGN.

X‐ray diffraction (XRD) and Raman analysis were performed to investigate the phase change during the precursor preparation steps, melamine, *α*‐Ni(OH)_2_‐Mel, and *β*‐Ni(OH)_2_‐Mel during the precursor preparation steps (Figure 1b,c and Figure S1a,b, Supporting Information). Characteristic peaks of melamine appeared in diffraction patterns of all the three samples (joint committee on powder diffraction standards (JCPDS) 39‐1950), indicating that melamine maintained its original structure during the precursor preparation (Figure S1a, Supporting Information). Enlarged XRD patterns in the range from 32° to 70° exhibited the diffraction peaks of nickel‐related species, excluding the characteristic peaks of melamine (Figure 1b).^[^ ^31^ ^]^ As a result of the reaction between nickel nitrate and NaOH, the characteristic peaks of *α*‐Ni(OH)_2_ were observed at 33.8° and 60.3° in *α*‐Ni(OH)_2_‐Mel, which coincided with the (101) and (110) planes, respectively (JCPDS 22‐0444). After the aging step, *β*‐Ni(OH)_2_‐Mel exhibited different characteristic peaks at 33.7°, 38.8°, 52.8°, and 59.7°, which were assigned to (100), (101), (102), and (110) planes of *β*‐Ni(OH)_2_, respectively (JCPDS 14‐0117).^[^ ^32^ ^]^ These results corresponded to the additional experimental with the same procedure but without melamine (Figure S2a,b, Supporting Information), further demonstrating the phase transition from *α*‐Ni(OH)_2_ to *β*‐Ni(OH)_2_ in the final *β*‐Ni(OH)_2_‐Mel.

Consistent with the XRD results, the Raman spectra exhibited that the characteristic peaks of melamine were preserved (Figure S1b, Supporting Information). As shown in the magnified Raman spectra in the range of 3500–3700 cm^–1^ (Figure 1c), the surface *ν*(OH) was dominant in the *α*‐Ni(OH)_2_‐Mel spectrum, while the *β*‐Ni(OH)_2_‐Mel spectrum exhibited only bulk *ν*(OH).^[^ ^28^ ^]^ The changes in the Raman spectra indicate that water molecules and polyatomic anions were removed from the interslab of Ni(OH)_2_ after the aging step, resulting in phase transition. In the same experiment but without melamine, the characteristic peak corresponding to NO_3_^–^ in the range of 1000–1100 cm^–1^ vanished as the NO_3_^–^ anions were removed after the phase transition to *β*‐Ni(OH)_2_ (Figure S2c,d, Supporting Information).^[^ ^33^ ^]^ However, it is noteworthy that the NO_3_^–^ characteristic peak remained in the *β*‐Ni(OH)_2_‐Mel spectrum, signifying that the NO_3_^–^ ions that escaped from the interslab were bound to melamine.

The surface chemical properties of melamine and *β*‐Ni(OH)_2_‐Mel were investigated by X‐ray photoelectron spectroscopy (XPS) measurements (Figure 1d and Figure S3, Supporting Information). The C 1s spectra of the melamine and *β*‐Ni(OH)_2_‐Mel were fitted into two main peaks at 284.7 and 287.7 eV, which are assigned to the adventitious graphitic carbon (C_ad_) and three‐coordinated carbon (C_3N_) in the triazine unit, respectively (Figure S3a,b, Supporting Information).^[^ ^31^ ^]^ In addition, Ni 2p spectrum analysis confirmed that *β*‐Ni(OH)_2_ was formed in *β*‐Ni(OH)_2_‐Mel (Figure S3c, Supporting Information). As shown in the deconvoluted N 1s spectrum of the *β*‐Ni(OH)_2_‐Mel, two additional peaks of —N^+^H═ and NO_3_^–^ were included in addition to the characteristic peaks corresponding to —NH_2_ and —N═ arising from melamine.^[^ ^31^ ^]^ This result, along with the Raman analysis, proves that the NO_3_^–^ ions, which escaped from the interslab during the aging step, protonated melamine and formed cross‐links for the subsequent heat treatment.

### 2.2 Formation Mechanism of Ni@N‐IGN during Heat Treatment

Thermogravimetric analysis (TGA) was performed under N_2_ atmosphere to observe the thermal behavior of *β*‐Ni(OH)_2_‐Mel during carbonization (Figure S4, Supporting Information). In contrast to melamine, which was completely decomposed above 300 °C, two inflection points were observed at 330 and 440 °C in the TGA curve of *β*‐Ni(OH)_2_‐Mel. We prepared heat‐treated samples at different temperatures based on the TGA curve to comprehensively investigate the carbonization process of *β*‐Ni(OH)_2_‐Mel (denoted as *β*‐Ni(OH)_2_‐Mel_*X*; *X* refers to heat treatment temperature). At 320 °C, before reaching the first inflection point, XRD and Raman analysis revealed the same diffraction pattern and spectrum as that of *β*‐Ni(OH)_2_‐Mel, respectively. This indicates that the original structure of *β*‐Ni(OH)_2_‐Mel was retained when melamine decomposed and Ni(OH)_2_ melted (Figure S5a,b, Supporting Information). In addition, a transmission electron microscopy (TEM) image showed that *β*‐Ni(OH)_2_‐Mel_320 existed in the form of amorphous aggregates (Figure S5c, Supporting Information).

**Figure 2:**
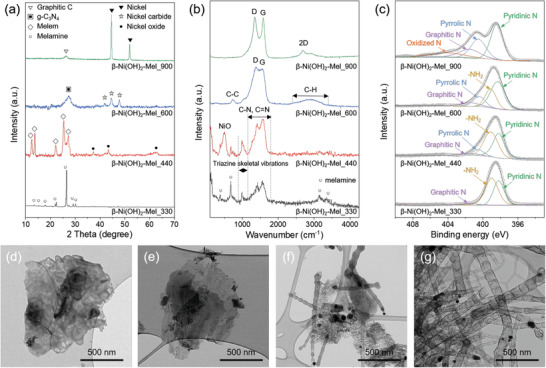
a) XRD patterns, b) Raman spectra, and c) XPS N 1s spectra of *β*‐Ni(OH)_2_‐Mel_330, 440, 600, and 900. TEM images of *β*‐Ni(OH)_2_‐Mel at different temperatures of d) 330, e) 440, f) 600, and g) 900 °C.

**Figure** **2**a exhibits the change in the XRD patterns of *β*‐Ni(OH)_2_‐Mel above the first inflection point. At the first inflection point (330 °C) in the TGA curve, the diffraction peaks of melamine decreased due to condensation of melamine, while the peaks corresponding to *β*‐Ni(OH)_2_ almost disappeared. The pattern of *β*‐Ni(OH)_2_‐Mel_440, at the second inflection point, demonstrated the formation of melem and NiO (JCPDS 71‐1179).^[^ ^32^, ^34^ ^]^ In case of *β*‐Ni(OH)_2_‐Mel_600 above the second inflection point, it can be noted that graphitic carbon nitride (g‐C_3_N_4_, JCPDS 87‐1526) was formed by polymerization of melem, while NiO was reduced to nickel carbide (JCPDS 01‐072‐1467).^[^ ^34^, ^35^ ^]^ At a higher temperature of 900 °C, the characteristic peaks of metallic nickel (JCPDS 04‐0850) appeared at 44.5° and 52° for the (111) and (200) planes, respectively, as nickel carbide was completely reduced. In addition, the diffraction peak located at 26.5° certified the formation of the graphitic structure (JCPDS 75‐1621).^[^ ^36^ ^]^

The Raman spectra of the samples at different temperatures further clarified the formation mechanism during the heat treatment (Figure 2b). For *β*‐Ni(OH)_2_‐Mel_330, the Raman spectrum exhibited a triazine skeletal vibration peak at 1000 cm^–1^ and C—N/C═N stretching in the range of 1170–1740 cm^–1^, confirming the condensation of melamine.^[^ ^37^ ^]^ At the second inflection point (440 °C), the triazine skeletal vibration peak and C—N/C═N stretching became sharp, while the melamine characteristic peaks vanished due to the complete polymerization of melamine to form melem. In addition, the NiO peak was observed at 500 cm^–1^, which is in agreement with the XRD results.^[^ ^38^ ^]^ The spectrum of *β*‐Ni(OH)_2_‐Mel_600 verified the formation of g‐C_3_N_4_ by the characteristic peaks located at 700, 1368, 1560, and 3000 cm^–1^, which are assigned to C—C, D‐, G‐band, and C—H, respectively.^[^ ^39^ ^]^ The formation of g‐C_3_N_4_ was further confirmed by Fourier transform infrared (FT‐IR) spectrum (Figure S6, Supporting Information). FT‐IR spectrum of *β*‐Ni(OH)_2_‐Mel_600 showed characteristic peaks between 700 and 1800 cm^–1^, which are ascribed to the trigonal N(C)_3_/bridging HN(C_2_) units and heptazine ring.^[^ ^40^ ^]^ For the final sample at 900 °C, sharp G and 2D peaks in the spectrum can be evidence of graphitic structure with high crystallinity.

The XPS measurements were performed to investigate the change in the chemical bonding state of the samples with temperature (Figure 2c and Figure S7, Supporting Information). The C 1s spectrum of the four samples showed an increasing tendency for C═C and a decreasing tendency for C_3N_ according to the heat treatment temperature. For the final sample heat‐treated at 900 °C, the C 1s spectrum exhibited a dominant C═C peak at 284.5 eV with peaks arising from surface functionalities such as C—N, C═N, and C‐O at 285.8, 287.1, and 290 eV, respectively (Figure S7a, Supporting Information). In addition, the Ni 2p spectrum revealed a change in the nickel bonding state during carbonization,^[^ ^41^ ^]^ which is concurrent with the XRD results (Figure S7b, Supporting Information). As shown in Figure 2c, the N 1s spectrum of *β*‐Ni(OH)_2_‐Mel_330 was fitted into three main peaks at 398.2, 399.1, and 401.5 eV corresponding to pyridinic N, —NH_2_, and graphitic N, respectively, indicating the beginning of condensation of melamine.^[^ ^31^ ^]^ For the sample at the second inflection point of 440 °C, the fitted N 1s spectrum exhibited additional peaks at 400.5 eV assigned to pyrrolic N. The condensation of melamine proceeded during the heat treatment, reducing the ratio of —NH_2_, while increasing the ratio of pyrrolic N and graphitic N. At the final temperature of 900 °C, *β*‐Ni(OH)_2_‐Mel_900 exhibited four peaks located at 398.2, 400.5, 401.6, and 404.1 eV, which can be attributed to pyridinic‐, pyrrolic‐, graphitic‐, and oxidized N, respectively.^[^ ^42^ ^]^

We further examined TEM to observe microstructural changes (Figure 2d–g). In the temperature range in which the condensation of melamine progressed, *β*‐Ni(OH)_2_‐Mel_330 and 440 appeared as amorphous aggregates, which is similar to that observed in the heat‐treated sample at 320 °C (Figure 2d,e). As shown in Figure 2f, spherical nickel carbide nanoparticles were observed when the temperature reached 600 °C. In particular, it can be noted that g‐C_3_N_4_ began to grow in an unusual 1D form around the nanoparticles. The morphological features of *β*‐Ni(OH)_2_‐Mel_600 maintained up to 800 °C (Figure S8, Supporting Information). At 900 °C, g‐C_3_N_4_ was decomposed to form a highly crystalline graphitic structure and reduce nickel carbide to nickel, thereby completing a uniform and unprecedented carbon nanostructure (Figure 2g).

### 2.3 Structural Features Imparting Crucial Functions to Ni@N‐IGN for the EM and EC Barriers

**Figure 3:**
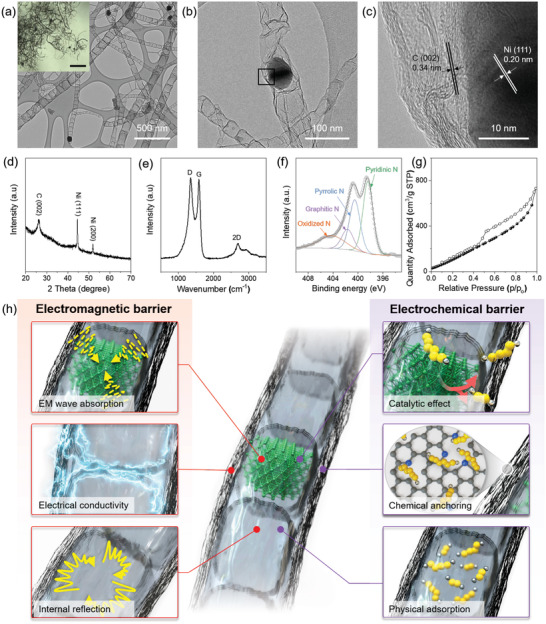
a–c) TEM images (inset of (a): OM image, scale bar: 20 µm), d) XRD pattern, e) Raman spectrum, f) XPS N 1s spectrum, and g) N_2_ adsorption/desorption isotherm of Ni@N‐IGN (inset of (g): pore size distribution). h) Schematic illustration of Ni@N‐IGN as a new class of carbon nanostructures for the EM and EC barriers.

An additional etching process was conducted on the final sample pyrolyzed at 900 °C to remove nonencapsulated nickel particles to obtain Ni@N‐IGN. High‐resolution TEM was performed to observe the microstructure of the as‐synthesized Ni@N‐IGN (**Figure** **3**a–c). Ni@N‐IGN showed uniformly synthesized 1D nanostructures, which is an assembly of graphitic nanocubes interconnected linearly with partly encapsulated nickel nanoparticles inside the nanocube (Figure 3a,b). Elemental distribution in Ni@N‐IGN was confirmed by energy‐dispersive spectroscopy (EDS) analysis equipped in TEM (Figure S9, Supporting Information). In addition, the optical microscopy (OM) image further confirmed homogeneous structure of Ni@N‐IGN with a high aspect ratio (Inset of Figure 3a). The high‐resolution TEM image revealed nickel nanoparticles with lattice fringes with a d‐spacing of 0.20 nm corresponding to the (111) crystal plane of Ni (Figure 3c). Moreover, it was observed that the wall of the nanocube was composed of thin layers with a d‐spacing of 0.34 nm, which can be attributed to the *c*‐axis of the graphitic sheets.^[^ ^43^ ^]^

As shown in Figure 3d, the XRD pattern of Ni@N‐IGN showed diffraction peaks at 26.4° corresponding to the (002) plane of graphitic carbon. In addition, the characteristic peaks at 44.5° and 51.9° were ascribed to the (111) and (200) planes of the Ni nanoparticles, respectively, which were embedded inside the nanocubes.^[^ ^44^ ^]^ The nickel content in Ni@N‐IGN was determined by TGA under an air atmosphere (Figure S10, Supporting Information). The original content of Ni was calculated to be 12.07 wt%, considering the oxidation of Ni. Raman spectroscopy characterized the degree of disorders in the carbon structure (Figure 3e). The sharp D band represented the distorted carbon structure caused by the nitrogen functionalities implanted in the graphitic structure. Nevertheless, it can be confirmed that Ni@N‐IGN possessed highly crystalline graphitic ordering through sharp and clear G‐ and 2D bands. Figure 3f shows the N 1s spectrum of Ni@N‐IGN to clarify the nitrogen bonding state. The N 1s spectrum was fitted to four main peaks located at 398.2, 400.5, 401.6, and 404.1 eV, originating from pyridinic‐, pyrrolic‐, graphitic‐, and oxidized N, respectively. Notably, Ni@N‐IGN contained 3.85 at% of the total nitrogen functionalities in the carbon nanostructure. The textural properties of Ni@N‐IGN were quantitatively analyzed using a nitrogen isotherm at 77 K, as shown in Figure 3g. Ni@N‐IGN possessed a high Brunauer–Emmett–Teller specific surface area of 278.32 m^2^ g^–1^. Figure S11 in the Supporting Information manifests the narrow pore size distribution of Ni@N‐IGN in the range from 3 to 4 nm. The high surface area and pore structure can be attributed to the wall of graphitic nanocube, which was composed of thin graphitic layers. In addition, these mesopores in the wall act as a passage to the hollow interior of the nanocube.

Figure 3h summarizes the advantageous features of an unprecedented nanostructure of Ni@N‐IGN. Three major structural characteristics of Ni@N‐IGN were presumed to achieve high performance in both the EM and EC barriers: i) The nickel nanoparticles embedded in the nanocube is a promising EM wave‐absorbing material due to large saturation magnetization and high permeability. In addition, it can serve as a catalyst to promote sluggish reaction in the Li–S batteries, thereby enhancing the reaction kinetics for high‐rate capability. ii) A highly ordered graphitic wall can provide a stable electrical pathway for effective EM wave absorption through conduction loss. The nitrogen functionalities inserted in the graphitic structure not only enhance the electrical conductivity but also improve the chemical interaction with soluble LiPSs to prevent the notorious shuttle reaction. iii) The graphitic nanocube, which is the basic unit of the entire nanostructure, provides empty meso‐ and macroporous space inside the nanocube. The hollow structure of Ni@N‐IGN induces internal reflection of EM radiation. Furthermore, the graphitic nanocube accommodates and confines LiPSs inside the hollow interior, thereby functioning as an EC barrier for the Li–S batteries. Together with these favorable features, we postulated that Ni@N‐IGN can achieve outstanding performance when evaluated as the EM and EC barriers.

### 2.4 Electromagnetic Measurements of Ni@N‐IGN

**Figure 4:**
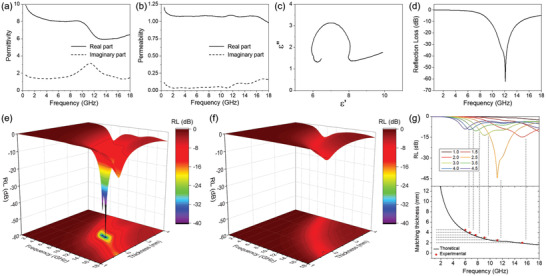
Real and imaginary a) permittivity and b) permeability of the Ni@N‐IGN and paraffin composite. c) Cole–Cole plot of the Ni@N‐IGN composite. d) RL value of the Ni@N‐IGN composite at a thickness of 2.45 mm. 3D plots of the RL curves of e) the Ni@N‐IGN composite and f) a‐Ni@N‐IGN composite. g) RL curves at different thicknesses and calculation matching thicknesses and peak frequency of the Ni@N‐IGN composite.

The EM barrier properties of Ni@N‐IGN were evaluated based on the reflection loss (RL) value obtained by the following equations^[^ ^14^, ^15^, ^45^, ^46^ ^]^

(1) $$Z_{\mathrm{in}}=Z_{0}\;{\left(\mu_{r}/\varepsilon_{r}\right)}^{1/2}\tanh\left[j\left(\frac{2\pi fd}{c}\right){\left(\mu_{r}\varepsilon_{r}\right)}^{1/2}\right]$$

(2) $$\mathrm{RL}\;\left(\mathrm{dB}\right)=\;20\log\left|\left(Z_{\mathrm{in}}-Z_{0}\right)/\left(Z_{\mathrm{in}}+Z_{0}\right)\right|$$

where *Z*_in_ is the characteristic impedance, *ε*_r_ (*ε*_r_ = *ε*′ − *jε*″) is the relative permittivity, *μ*_r_ (*μ*_r_ = *μ*′ − *jµ*″) is the relative permeability, *d* is the thickness of the sample, *Z*_0_ is the free space impedance (≈377 Ω), *f* is the frequency, and *c* is the velocity of microwave in free space. The permittivity and permeability were measured (**Figure** **4**a,b), and the RL value was obtained based on Equations (1) and (2). Because the permittivity and permeability of conducting materials cannot be directly measured, dielectric composite materials are prepared by mixing insulating paraffin and Ni@N‐IGN. The real and imaginary terms of permittivity of the Ni@N‐IGN composite are in the ranges of 6–10 and 1–3 at 0.5–18 GHz, respectively (Figure 4a). These values are appropriate for an EM wave‐absorbing film because a very high permittivity prevents impedance matching and causes low Z_in_ and RL values, while very low permittivity inevitably increases the film thickness. Interestingly, a small amount of Ni@N‐IGN (5 wt%) is enough to obtain appropriate permittivity due to the high aspect ratio of the material. For practical applications, the loading level of the filler should be low enough to decrease the cost and improve the processability. Compared with other EM wave‐absorbing films based on magnetic and carbon hybrid materials,^[^ ^14^, ^46^ ^]^ the Ni@N‐IGN‐based film can be designed in a more efficient manner.

It is known that the real terms of permittivity (*ε*′) and permeability (*μ*′) indicate EM wave storage and the imaginary terms of permittivity (*ε*″) and permeability (*μ*″) indicate that the EM wave dissipates during the propagation of the EM wave in the material. Interestingly, a tendency to decrease the real permittivity and increase the imaginary permittivity at ≈12 GHz was observed. Interestingly, a tendency to decrease the real permittivity from 10 to 12 GHz region was observed. On the other hand, the imaginary permittivity has a peak near 12 GHz. It is believed that those are attributed to the resonance induced by the interfacial polarization and multiple reflection in the porous structure.^[^ ^47^ ^]^ The interfacial polarization was observed because of charge accumulation at the interface between the Ni nanoparticles and graphitic nanocube.^[^ ^48^ ^]^ The polarization was verified by the formation of semicircles in the Cole–Cole plot based on the following equation^[^ ^49,50^ ^]^

(3) $${\left(\varepsilon^{\prime }-\frac{\varepsilon_{s}+\varepsilon_{\infty }}{2}\right)}^{2}+\;{\left(\varepsilon^{\prime \prime }\right)}^{2}={\left(\frac{\varepsilon_{s}-\varepsilon_{\infty }}{2}\right)}^{2}\;$$

where *ε*_s_ is the static dielectric constant and *ε*_∞_ is the dielectric constant at infinite frequency. Figure 4c shows the obvious semicircles in the plot and confirms that the interfacial polarization induced dielectric relaxation. This relaxation will be beneficial for the preparation of the EM wave‐absorbing film with high absorption property. The loss ability of the EM wave of the composite is represented by the dielectric loss (tan *δ_*ε*_*, tan *δ_*ε*_* = (*ε*″/*ε*′)) and magnetic loss (tan *δ_µ_*, tan *δ_µ_* = (*μ*″/*μ*′)). The influence of the dielectric relaxation is also observed in the dielectric loss tangent (Figure S12a, Supporting Information), indicating that the EM wave‐absorbing property is maximized at ≈12 GHz. The magnetic loss tangent of the composite is smaller than the dielectric loss tangent for all the frequency regions, indicating that the dielectric loss is dominant compared with the magnetic loss (Figure S12b, Supporting Information). The magnetic loss tangent of most EM wave‐absorbing films is smaller than the dielectric loss tangent in the gigahertz region because the magnetic property disappears at a high frequency above the ferromagnetic resonance frequency.^[^ ^51^ ^]^ Despite the low magnetic loss tangent, Equations (1) and (2) indicate that high permeability is desirable to obtain high *Z*_in_ and RL, and thus, incorporation of magnetic materials is crucial to prepare an EM wave‐absorbing film with a high RL value. The real and imaginary permeabilities of the Ni@N‐IGN composites are ≈1.07 and ≈0.05, respectively, for the whole X‐band (8–12 GHz) region (Figure 4b) due to the nickel nanoparticles embedded in the graphitic nanocubes. These high permeabilities can make a big difference in the EM wave‐absorbing property.

The RL values of the composite film with varying film thicknesses was calculated based on Equations (1) and (2), as shown in Figure 4e. As expected from the permittivity graph of the composite, an RL peak appears at ≈12 GHz, where the dielectric relaxation was observed. The optimum RL value of −62.1 dB at 12.1 GHz was obtained when the thickness was 2.45 mm (Figure 4d). We summarized the previous studies of EM wave absorption properties of carbon/magnetic metal hybrid materials (Table S1, Supporting Information). The Ni@N‐IGN‐based composite obviously has high reflection loss despite the small amount of the hybrid filler. It showed that the interface polarization indeed induced EM wave absorption in this system, and Ni@N‐IGN exhibited a proper structure for the EM wave‐absorbing material. The EM wave absorption of the composite can be evaluated by the quarter‐wavelength matching model described by the following equation^[^ ^14^ ^]^

(4) $$t_{m}=\frac{nc}{4f_{m}\sqrt{\left|\varepsilon_{r}\right|\left|\mu_{r}\right|}}\;\left(n\;=\;1,\;3,\;5,\;\ldots \right)$$

where *t*_m_ is the thickness of the EM wave‐absorbing film, *c* is the light velocity, and *f*_m_ is the corresponding frequency of the optimum RL value at the thickness. The correlation between the thickness and optimum RL value calculated from the quarter‐wavelength matching model are shown in Figure 4g. The value calculated from Equation (4) well matched with the value obtained from the RL curves, implying that the absorption behavior can be described using the quarter‐wavelength matching model. In particular, the absorption was optimized near the 12 GHz frequency at 2.45 mm thickness because the EM wave dissipated during the propagation in the composite due to the dielectric loss, and the thickness well matched with the quarter‐wavelength matching theory.

It is noteworthy that Ni nanoparticles incorporated in Ni@N‐IGN play a critical role in the EM wave absorption. We prepared a sample with less amount of Ni nanoparticles (2.73 wt%) by air oxidation and subsequent HCl etching of Ni@N‐IGN (denoted as a‐Ni@N‐IGN, Figure S13, Supporting Information). Contrast to the Ni@N‐IGN‐based film, the resonance near 12 GHz in the permittivity graph and a semicircle in the Cole–Cole plot were not observed in the a‐Ni@N‐IGN‐based film (Figure S14, Supporting Information). Moreover, permeability of the a‐Ni@N‐IGN‐based film became smaller since density of the magnetic Ni nanoparticles reduced. As shown in Figure 4f, the absorption peak near 12 GHz in the RL curves significantly reduced in the a‐Ni@N‐IGN‐based film, indicating that the magnetic property and resonance effect of Ni@N‐IGN significantly contribute to the EM wave absorption of the system. Consequently, the prominent EM wave‐absorption performance was promoted by three factors: 1) highly ordered graphitic wall and N‐doped carbon that led to conduction loss, 2) porous structure of Ni@N‐IGN promotes multiple internal reflection, and 3) embedding nickel nanoparticles in the pores not only induces interfacial polarization but also increases the permeability of the materials. These three factors of Ni@N‐IGN effectively integrated to exhibit a high RL value.

### 2.5 Adsorption Ability and Electrocatalytic Effect of Ni@N‐IGN

**Figure 5:**
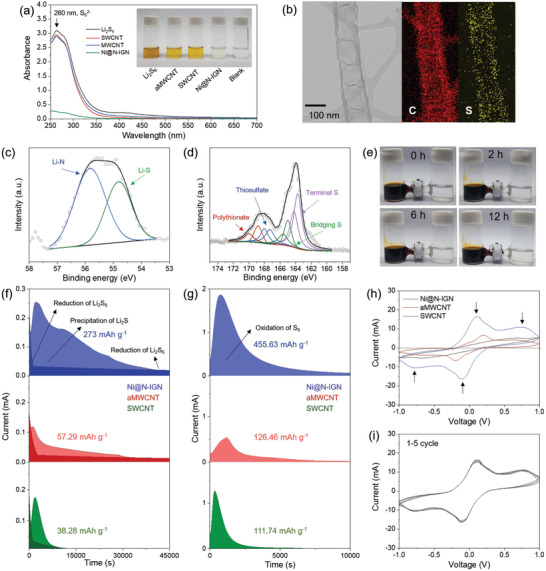
a) UV–vis spectra with different samples after the Li_2_S_6_ adsorption test (Inset of (a): digital photographs of Li_2_S_6_ solution after the adsorption test). b) TEM image and elemental maps of carbon and sulfur after the Li_2_S_6_ adsorption test. XPS c) Li 1s and d) S 2p spectra of Ni@N‐IGN after the Li_2_S_6_ adsorption test. e) Visual inspection of the Li_2_S_6_ diffusion experiment for the Ni@N‐IGN separator. Potentiostatic f) discharge profiles and g) charge profiles of different cells. h) CV curves of the different symmetric cells. i) CV curves of the Ni@N‐IGN symmetric cell for five cycles.

To assess the functional separator as an EC barrier for the Li—S batteries, a typical polypropylene (PP) separator was covered with Ni@N‐IGN through the conventional slurry casting method. It was confirmed that the Ni@N‐IGN layer was well positioned on the separator without delamination and cracking even after bending or punching (Figure S15a,b, Supporting Information). The cross‐sectional scanning electron microscopy image revealed that the Ni@N‐IGN layer covered the separator homogeneously with a thickness of 10.89 µm (Figure S15c, Supporting Information). Since Ni@N‐IGN has a 1D structure, the adsorption capabilities and electrocatalytic effect were compared with carbon nanotubes (CNTs) which are representative 1D carbon structures. Single‐walled carbon nanotube (SWCNT) and acid‐treated multiwalled carbon nanotube (aMWCNT) were chosen for analytical comparison. The solution with Ni@N‐IGN became transparent, almost equivalent to the blank solution, after the adsorption test, while the solutions containing the aMWCNT and SWCNT were still brown in color as the pristine Li_2_S_6_ solution (inset of **Figure** **5**a). Furthermore, UV–vis spectroscopy of the filtered solution showed significant differences in the absorbance at 250–300 nm related to Li_2_S_6_,^[^ ^52^ ^]^ thereby confirming the superior adsorption ability of Ni@N‐IGN compared to the aMWCNT and SWCNT (Figure 5a).

EDS analysis equipped in TEM was performed to investigate the distribution of sulfur species within the samples after the Li_2_S_6_ adsorption test. As shown in Figure 5b, the elemental mapping of Ni@N‐IGN revealed that sulfur species were adsorbed on both the surface and inside of Ni@N‐IGN. This is because the surface chemical interaction with LiPS was improved by the nitrogen functionalities as well as the hollow interior can be used as an LiPS reservoir.^[^ ^53^, ^54^, ^55^ ^]^ In contrast, the aMWCNT was found to adsorb trace amounts of sulfur species due to a few functional groups derived from the acid treatment (Figure S16a, Supporting Information). In the case of the SWCNT without the functional group on the surface, sulfur adsorption did not occur (Figure S16b, Supporting Information).

To further unveil the bonding state of Ni@N‐IGN after the Li_2_S_6_ adsorption, the XPS analysis was performed on the separated material. The Li 1s spectrum was fitted into two peaks located at 54.7 and 55.7 eV corresponding to the Li—S and Li—N bonds,^[^ ^56^ ^]^ respectively, which can be attributed to the interaction between LiPS and Ni@N‐IGN (Figure 5c). In addition, four sulfur bonding states appeared in the S 2p spectrum (Figure 5d). The peaks assigned to the terminal S and bridging S of Li_2_S_6_ appeared at 163.8 and 164.9 eV, respectively, which are positive‐shifted values, originating from the interaction between Ni@N‐IGN and sulfur. Moreover, four peaks were observed at 167.3, 168.1, 168.8, and 169.9 eV, corresponding to the thiosulfate and polythionate states obtained through the surface redox reaction.^[^ ^57^ ^]^

The brown Li_2_S_6_ solution and the transparent blank solution were separated by a modified separator to visualize the LiPS blocking ability. In case of the Ni@N‐IGN separator, the right side of H‐type cell remained transparent even after 12 h, demonstrating the effectiveness of the Ni@N‐IGN separator for blocking the diffusion of LiPSs (Figure 5e). On the contrary, it was observed that the color of the blank solution changed quickly as the aMWNCT and SWCNT separator could not prevent the passage of LiPS through the separator (Figure S17, Supporting Information).

It is a vital issue to improve the sluggish kinetics of the conversion reaction from soluble Li_2_S*_x_* (4 ≤ *x* ≤ 8) to insoluble Li_2_S.^[^ ^58^ ^]^ The potentiostatic discharge experiment was performed to explore the electrocatalytic effect on the conversion reaction. As shown in Figure 5f, Ni@N‐IGN showed a peak current of 0.25 mA, which is higher than the aMWCNT (0.11 mA) and SWCNT (0.17 mA), indicating the faster kinetics for the Li_2_S nucleation.^[^ ^59^ ^]^ In addition, the electrode deposition capacity was measured using Faraday's law as 273, 57.29, and 38.28 mAh g^–1^ for the Ni@N‐IGN, aMWCNT, and SWCNT, respectively. These results verified that the reaction kinetics of Li_2_S precipitation was significantly improved by the catalytic effect of Ni@N‐IGN. To clarify the contribution of Ni nanoparticles to the catalytic effect, the potentiostatic discharge experiment was performed using the a‐Ni@N‐IGN cell with lower Ni content of 2.73 wt% (Figure S18, Supporting Information). The electrode deposition capacity significantly reduced to 73.47 mAh g^–1^. This result verified that the catalytic effect is dominantly attributed to Ni nanoparticles embedded in graphitic nanocubes. Furthermore, the potentiostatic charge experiments at 2.4 V were conducted to study the electrocatalytic effect on the solid–liquid phase transition during the reverse process (Figure 5g). As expected, Ni@N‐IGN exhibited a higher peak current (1.87 mA) as compared to the aMWCNT (0.54 mA) and SWCNT (1.26 mA). The capacity of Ni@N‐IGN (455.63 mAh g^–1^) corresponding to the oxidation of Li_2_S was also higher than the aMWCNT (126.46 mAh g^–1^) and SWCNT (111.74 mAh g^–1^). These results proved the outstanding electrocatalytic effect of Ni@N‐IGN on the precipitation of Li_2_S as well as the oxidation reaction of Li_2_S.

Cyclic voltammetry (CV) analysis of symmetric cells was conducted using an electrolyte containing Li_2_S_8_ to further validate the electrocatalytic effect on the conversion reaction.^[^ ^60^ ^]^ The redox peaks in the CV curve can be ascribed to the redox reaction of Li_2_S_8_, which is the sole electrochemical active species in the symmetric cell system. During the cathodic scan, the reduction and oxidation of the sulfur species occur at the working and counter electrodes, respectively, while it proceeds in the opposite direction in the anodic scan. As shown in Figure 5h, the CV curve of the Ni@N‐IGN symmetric cell displayed four pronounced redox peaks with a higher peak current compared to the aMWCNT and SWCNT symmetric cells. This is consistent with the potentiostatic experiments, demonstrating that the distinct nanostructures of Ni@N‐IGN can facilitate the sluggish conversion reaction to improve the kinetics of the Li–S batteries. Moreover, for the Ni@N‐IGN symmetric cell, the CV curve was retained without any noticeable change in the five cycles, indicating the stability of the resulting material during the electrochemical process (Figure 5i).

### 2.6 Electrochemical Performance of Li–S Cells with the Ni@N‐IGN Separator

**Figure 6:**
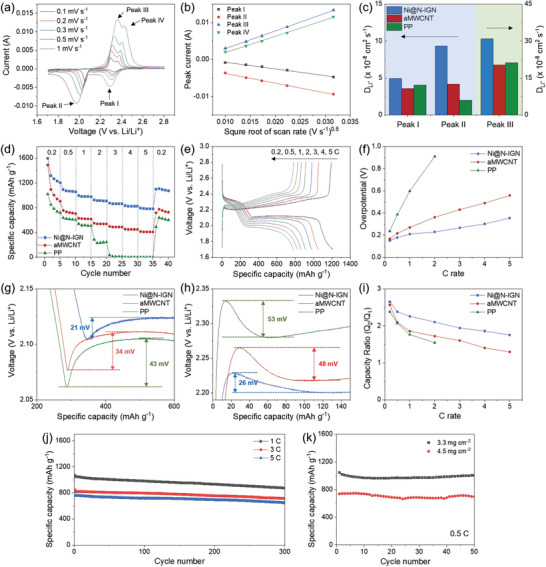
a) CV curves of the Li–S cell with the Ni@N‐IGN separator at various scan rates from 0.1 to 1 mV s^–1^. b) CV peak currents versus the square root of the scan rate plots for the three characteristic peaks of the Ni@N‐IGN separator cell. c) Diffusion coefficient of the different cells. d) Rate performance of the Li–S cells with different separators at various C rates from 0.2 to 5 C. e) Charge/discharge voltage profiles of the Ni@N‐IGN separator cell at various C rates from 0.2 to 5 C. f) Overpotential values, enlarged view of the g) discharge, h) charge voltage profiles, and i) *Q*_2_/*Q*_1_ ratio at various C rates of the different cells. j) Cycling performance for extended cycles at 1, 3, and 5 C of the cells with the Ni@N‐IGN separator. k) Cycling performance of the Ni@N‐IGN separator cell at 0.5 C with higher sulfur loading conditions.

To evaluate the electrochemical performance of Ni@N‐IGN as an EC barrier, a 2032 type coin cell was fabricated with the Ni@N‐IGN separator and a conventional Ketjen Black‐sulfur (KB‐S) electrode. As shown in **Figure** **6**a, the CV curve showed two clear reduction peaks in the cathodic scan and two oxidation peaks in the anodic scan. Peaks I and II refer to the stepwise reduction of S_8_ into LiPS (Li_2_S*_x_*, 4 ≤ *x* ≤ 8) and Li_2_S, respectively. For the opposite direction, peaks III and IV were assigned to the stepwise oxidation.^[^ ^61^ ^]^ In addition, obvious redox peaks were preserved even with a high scan rate, representing stable electrochemical reversibility of the Ni@N‐IGN separator. In contrast, for the aMWCNT and a typical PP separator (Figure S19, Supporting Information), the redox peaks became broad and vanished with increasing the scan rate. In addition, peaks III and IV were merged into one peak due to the sluggish kinetics of the redox reactions. Since peak IV disappeared in the aMWCNT and PP separator cells, peaks I, II, and III were further analyzed. The Ni@N‐IGN separator exhibited a higher peak current for three redox peaks at high scan rates than the aMWCNT and PP separator (Figure S20, Supporting Information). Furthermore, the Ni@N‐IGN separator possessed the highest cathodic peak potentials and the lowest anodic peak potentials (Figure S21, Supporting Information). Detailed information obtained from the CV curve is evidence of the electrocatalytic effect of Ni@N‐IGN, which accelerates the redox reaction and alleviates cell resistance. The relationship between the peak current (*I_P_*) and scan rate (*v*) can be described according to the Randles–Sevcik equation.^[^ ^62^ ^]^

(5) $$I_{P}=\;2.69\times 10^{5}n^{1.5}AD^{0.5}Cv^{0.5}$$

Here, *n*, *A*, *D*, and *C* are the electron transfer number (*n* = 2 for Li–S chemistry), electrode area, Li^+^ ion diffusion coefficient, and Li^+^ ion concentration, respectively. A linear relationship was confirmed between the peak current and square root of scan rate, which is indicative of a diffusion‐limited process (Figure 6b and Figure S22, Supporting Information). Figure 6c represents the Li^+^ ion diffusion coefficient of three redox peaks, determined by the above relationship. Diffusion coefficient is a descriptor of reaction kinetics, mainly influenced by the LiPS viscosity in the electrolyte.^[^ ^63^ ^]^ It can be observed that the Ni@N‐IGN separator cell exhibited the highest Li^+^ ion diffusivity for the overall redox reaction. This is because LiPS was consumed in the electrolyte by the adsorption or rapid conversion reaction, which validates the superior functions of Ni@N‐IGN as an EC barrier.

Galvanostatic charge/discharge measurement was conducted at various current rates from 0.2 to 5 C to evaluate the electrochemical performance of the Ni@N‐IGN separator (1 C = 1672 mA g^–1^). As expected, the Ni@N‐IGN separator cell exhibited a significantly improved specific capacity, rate capability, and stable Coulombic efficiency close to 100% (Figure 6d and Figure S23 and Table S2, Supporting Information). The Ni@N‐IGN separator cell delivered the highest average specific capacity of 1323, 1069, 998, 914, 866, 826, and 785 mAh g^–1^ at 0.2, 0.5, 1, 2, 3, 4, and 5 C, respectively. In addition, the specific capacity recovered to 1106 mAh g^–1^ after the multirate test, indicating the electrochemical reversibility of the Ni@N‐IGN separator cell. The voltage profiles were further analyzed to understand the origin of performance improvement. During the discharge process at 0.2 C, two plateaus were identified in the voltage profile, originating from the multistep reaction of sulfur (Figure S24, Supporting Information). In the reverse process, the long plateau in the charge process was attributed to the stepwise oxidation process of Li_2_S to S_8_.^[^ ^64^ ^]^ The Ni@N‐IGN separator cell showed the highest capacity at 0.2 C, indicating high sulfur utilization due to the adsorption ability of Ni@N‐IGN to confine soluble LiPSs on the cathode. After five cycles at 0.2 C, the cell was disassembled and the Ni@N‐IGN separator was washed with 1,3‐dioxolane (DOL)/dimethoxyethane (DME) for postmortem characterization. XRD patterns confirmed that the nickel embedded in the graphitic nanocube preserved its original crystal structure, which is indicative of phase stability during electrochemical reactions (Figure S25, Supporting Information). As shown in Figure 6e and Figure S26 in the Supporting Information, the voltage profiles varied as the C rate increased. In the case of the cell with the PP separator, the reaction plateaus vanished remarkably at high C rates above 1 C. In contrast, the Ni@N‐IGN separator cell maintained the reaction plateaus in the voltage profile even at 5 C, revealing outstanding rate capability. When estimating the overpotential by the difference in the lower discharge and charge plateaus, the overpotential of the PP cell increased rapidly with the increasing C rate (Figure 6f). As the aMWCNT separator can function as an upper current collector to compensate the electrical conductivity, overpotential was reduced moderately in the aMWCNT separator cell. Noticeably, the Ni@N‐IGN separator further reduced the overpotential, resulting from its exceptional function as an EC barrier confining active materials and promoting the conversion reaction.

A closer view of the discharge voltage profile shows a voltage dip between the two plateaus, which is a small overpotential caused by the nucleation of insoluble lithium sulfide.^[^ ^65^ ^]^ As shown in Figure 6g, the Ni@N‐IGN separator cell showed the smallest voltage dip of 21 mV during the discharge, while the aMWCNT and PP separator cell showed 34 and 43 mV, respectively. This demonstrates the facilitated conversion reaction from soluble LiPS into insoluble lithium sulfide for Ni@N‐IGN. Furthermore, activation energy is required to activate the insulating Li_2_S into Li_2_S*_x_* in the initial part of the charging process, resulting in a large voltage hill (Figure 6h).^[^ ^66^ ^]^ The Ni@N‐IGN separator cell showed a voltage hill of 26 mV, which was lower than the aMWCNT and PP separator cell. This closer inspection of the voltage profile further verifies the significant enhancement of the reaction kinetics in both the reaction directions.

The specific capacity was divided by the upper plateau capacity (*Q*_1_) and lower plateau capacity (*Q*_2_) for the quantitative analysis of the conversion reaction (Figure S27, Supporting Information). The ratio of *Q*_2_/*Q*_1_ is an indicator of the conversion rate of sulfur, which would be 3 if the conversion reaction is theoretically completed during discharge. The Ni@N‐IGN separator cell showed a larger ratio value of 2.65 at 0.2 C than those of the aMWCNT and PP separator cell (Figure 6i). A higher *Q*_2_/*Q*_1_ ratio at a lower C rate is attributed to the adsorption ability of Ni@N‐IGN, which restricts soluble LiPS on the cathode side. Furthermore, the Ni@N‐IGN separator cell maintained a higher *Q*_2_/*Q*_1_ ratio even at 5 C, signifying its superior electrocatalytic effect for the conversion reaction.

The Ni@N‐IGN separator cell was tested at high rates of 1, 3, and 5 C for extended cycles (Figure 6j and Table S3, Supporting Information). The Ni@N‐IGN separator cell exhibited initial discharge capacities of 1067, 850, and 814 mAh g^–1^ and preserved 874, 714, and 644 mAh g^–1^ after 300 cycles at 1, 3, and 5 C, respectively. The capacity decays were 0.06%, 0.05%, and 0.07% per cycle with a high Coulombic efficiency of ≈99% (Figure S28a, Supporting Information), manifesting stable cycling performance for prolonged cycles. Furthermore, to evaluate the practicality for industrial application, we examined the electrochemical performance of the Ni@N‐IGN separator cell with high sulfur loading (Figure 6k and Table S4, Supporting Information). The Ni@N‐IGN separator cell exhibited a high specific capacity stabilized at ≈1000 and 700 mAh g^–1^ at 0.5 C with higher sulfur loading of 3.3 and 4.5 mg cm^–1^, respectively, while maintaining a high Coulombic efficiency above 95% during cycling (Figure S28b, Supporting Information). This clarifies the stable cycling performance of the Ni@N‐IGN separator cells even with high sulfur loading conditions.

## 3 Conclusion

In summary, we developed novel interconnected graphitic nanocubes with partly embedded by nickel nanoparticles derived from the coordinative compound between melamine and nickel ion through phase transition and the subsequent carbonization process. Comprehensive characterization and evaluation of Ni@N‐IGN manifested three major factors that can be attributed to the outstanding performance of EM and EC barriers: 1) The meso‐ and macroporous space of the hollow graphitic nanocube induced multiple internal reflection of EM waves and confined soluble LiPSs as an EC barrier. 2) N‐doped highly ordered graphitic wall promoted conduction loss of the EM wave and enhanced chemical anchoring of soluble LiPSs. 3) Nickel nanoparticles in the graphitic nanocube improved the permeability and encouraged interfacial polarization as the EM barrier, while functioned as an electrocatalyst to improve the sluggish kinetic of Li–S chemistry. As a consequence, the Ni@N‐IGN‐based film as an EM barrier exhibited an optimum RL value of −62.1 dB at 12.1 GHz even with a small amount of Ni@N‐IGN. Furthermore, the resulting cells with a Ni@N‐IGN separator as the EC barrier exhibited outstanding rate capabilities (998 and 785 mAh g^–1^ at 1 and 5 C, respectively) and stable cycling performance at higher C rates. Taking into account the multifarious outstanding performances of the resulting carbon nanostructure, we believe that the presented approach will provide a broad alternative toward designing novel carbon nanomaterials possessing distinct microstructures and versatile functions.

## 4 Experimental Section

### Synthesis of Ni@N‐IGN

Here, 1.37 g of melamine (Sigma‐Aldrich) was dissolved in 40 mL of distilled water. Then, 0.42 g of nickel nitrate hexahydrate (Sigma‐Aldrich) and 0.3 g of sodium hydroxide (Ducksan Pure Chemicals) were added to the solution. The reaction mixture was dispersed for 5 min by ultrasonication to obtain a uniform dispersion. The dispersion was centrifuged at 9000 rpm for 10 min to remove the residual ions. The precipitate was aged in 40 mL of distilled water for 5 h at 85 °C. The mixture was dried to obtain a light‐green powder. The powder was placed in a box furnace and heat‐treated for 3 h at 900 °C under N_2_ atmosphere at a heating rate of 5 °C min^–1^. The resulting material was etched with 1 m hydrochloric acid for 3 h at 80 °C to remove the noncapsulated nickel particles.

### Characterization

XRD patterns were recorded using an X‐ray diffractometer (G.N.R. Europe 600) with a Ni beta‐filtered Cu *K*_*α*_ radiation (*λ* = 0.154 nm). Raman spectroscopy was employed with a RAMAN plus confocal laser Raman microscope (Ramanforce, Nanophoton) to analyze the surface physicochemical structure of the products with a laser wavelength of 532 nm. XPS (K‐Alpha, Thermo Fisher) was performed to observe the changes in the chemical composition at each step. TGA (TGA‐N1000, Sinco) was performed from 25 to 900 °C under N_2_ atmosphere and air atmosphere at a heating rate of 5 °C min^–1^. TEM (JEM‐2100F, JEOL) was utilized to observe the microstructures of the samples.

### Electromagnetic Measurements

The EM characteristics of Ni@N‐IGN and paraffin composite specimen were measured using a vector network analyzer (Agilent N5230A) and a 7 mm coaxial airline at frequencies ranging from 0.5 to 18 GHz. The samples were prepared by physically mixing 5 wt% of Ni@N‐IGN and 95 wt% of paraffin and subsequent pressing into a toroidal shape (outer and inner diameters were 7 and 3.04 mm, respectively). The complex permittivity and permeability were computed from the measured s‐parameters based on the Nicholson‐Ross‐Weir (NRW) theoretical calculations.

### Preparation of the Ni@N‐IGN Separator

A typical PP separator (Celgard 2400) was coated with Ni@N‐IGN using the slurry casting method. The slurry was composed of Ni@N‐IGN, Super P, and polyvinylidene fluoride (PVDF) in a weight ratio of 8:1:1 and mixed in *N*‐methyl‐2‐pyrrolidone (NMP) as a dispersant. The slurry was coated on one side of the PP separator using a doctor blade and dried at 60 °C under vacuum overnight.

### Preparation of the Li_2_S_6_ and Li_2_S_8_ Solutions for Electrochemical Analysis

Sulfur (Sigma‐Aldrich) and Li_2_S (99.98%, Sigma‐Aldrich) were added to DOL (Sigma‐Aldrich) and DME (Sigma‐Aldrich) (1:1, volume ratio) and stirred overnight at 60 °C until the solution turned to dark brown. The molar ratios of sulfur and Li_2_S were controlled as 5:1 and 7:1 for Li_2_S_6_ and Li_2_S_8_ solutions, respectively. The active materials (Ni@N‐IGN, aMWCNT, and SWCNT) were mixed with Super P and PVDF in a weight ratio of 8:1:1 in NMP. Then, the slurry was coated onto an aluminum foil and dried overnight at 60 °C under vacuum. For the symmetric cell test, the same two electrodes were used as the working and counter electrodes. DOL/DME (1:1, volume ratio) containing 0.5 m Li_2_S_8_ and 1 m lithium bis(trifluoromethane sulfonyl) imide (LiTFSI, Sigma‐Aldrich) was used as an electrolyte. For the Li_2_S nucleation test, the electrodes (Ni@N‐IGN, aMWCNT, and SWCNT) were used as the working electrode and lithium foil was used as both the counter and reference electrode. Lithium metal thickness was 0.7 mm and area was 2.01 cm^2^. Then, 25 µL of 0.5 m Li_2_S_8_ with 1 m LiTFSI in DOL/DME was used as the catholyte, and 20 µL of 1 m LiTFSI solution without Li_2_S_8_ was used as the anolyte. The cells were first discharged galvanostatically at 0.05 C to 2.12 V and were potentiostatically discharged at 2.11 V until the current decreased to 10^−5^ A. For the Li_2_S dissolution test, the cells after the first discharge to 1.7 V were charged to 2.3 V at 0.05 C and were potentiostatically charged at 2.4 V until the current decreased to 10^–5^ A.

### Electrochemical Measurements

Ketjen Black (EC 300J, Mitsubishi chemical) and sulfur powder (Sigma‐Aldrich) were physically mixed in a weight ratio of 7:3 and then heated to 155 °C overnight. The KB‐S cathode was prepared using the slurry coating method with a mixture of KB‐S (80 wt%), Super P (10 wt%), and CMC (carboxymethyl cellulose, 10 wt%) in distilled water. The slurry was coated on the aluminum foil and the electrode was dried under vacuum. The specific capacity was calculated based on sulfur mass. The typical sulfur loading for cathode was 1 mg cm^−2^ and electrode thickness was 30 µm. For the electrochemical test with the higher sulfur loading conditions, the slurry was prepared at a 7:2:1 weight ratio of sulfur composite, Super P, and CMC in distilled water, and the loading amount of sulfur was increased to 3.3 and 4.5 mg cm^−2^. The 2032 type coin cell was assembled with the KB‐S electrode and Ni@N‐IGN separator in an argon‐filled glove box (KK‐011Ms, Korea Kiyon). The electrolyte was prepared by dissolving 1 m LiTFSI in DOL/DME (1:1, volume ratio) with lithium nitrate (1 wt%, LiNO_3_, Sigma‐Aldrich). Then, galvanostatic discharge/charge tests were evaluated in a voltage window of 1.7–2.8 V versus Li/Li^+^ at various C rates (1 C = 1672 mA g^–1^) using a battery cycler at 30 °C (WBCS 3000, WonATech).

## Conflict of Interest

The authors declare no conflict of interest.

## Supporting information

Supporting Information

## Data Availability

The data that support the findings of this study are available from the corresponding author upon reasonable request.
