# A New Class of Carbon Nanostructures for High‐Performance Electro‐Magnetic and ‐Chemical Barriers

**DOI:** 10.1002/advs.202200055

**Published:** 2022-02-14

**Authors:** Jae Hui Park, Yun Ji Oh, Dong Yoon Park, Joonsik Lee, Jae Seo Park, Chong Rae Park, Jae Ho Kim, Taehoon Kim, Seung Jae Yang

*Adv. Sci*. **2021**, *8*, 2102718

DOI: 10.1002/advs.202102718

In the originally published version of the article, one grant number is incorrect in the Acknowledgements. Please find the correct Acknowledgements here:

