# Supplementary material for: A New Class of Carbon Nanostructures for High‐Performance Electro‐Magnetic and ‐Chemical Barriers
Source: Adv Sci (Weinh). 2021 Sep 30;8(22):2102718. doi: 10.1002/advs.202102718 (PMC8596133; doi:10.1002/advs.202102718)
Supplement: Supplementary file 1 — Supporting Information [file ADVS-8-2102718-s001.pdf]

© 2021 Wiley-VCH GmbH

ADVANCED SCIENCE
Open Access

Supporting Information

for *Adv. Sci.*, DOI: 10.1002/advs.202102718

A New Class of Carbon Nanostructures for High-Performance Electro-Magnetic and -Chemical Barriers

Jae Hui Park, Yun Ji Oh, Dong Yoon Park, Joonsik Lee, Jae Seo Park, Chong Rae Park, Jae Ho Kim, Taehoon Kim,* and Seung Jae Yang**

Supporting Information

A New Class of Carbon Nanostructures for High-Performance Electro-Magnetic and -Chemical Barriers

Jae Hui Park, Yun Ji Oh, Dong Yoon Park, Joonsik Lee, Jae Seo Park, Chong Rae Park, Jae Ho Kim, Taehoon Kim,* and Seung Jae Yang**

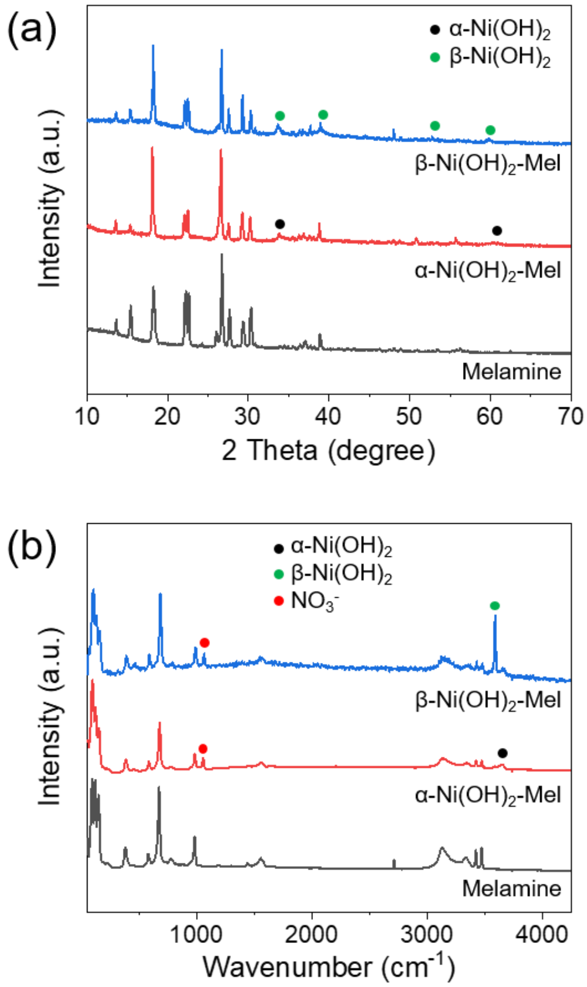

Figure S1. (a) XRD patterns and (b) Raman spectra of melamine, α-$Ni(OH)_2$-Mel, and β-$Ni(OH)_2$-Mel.

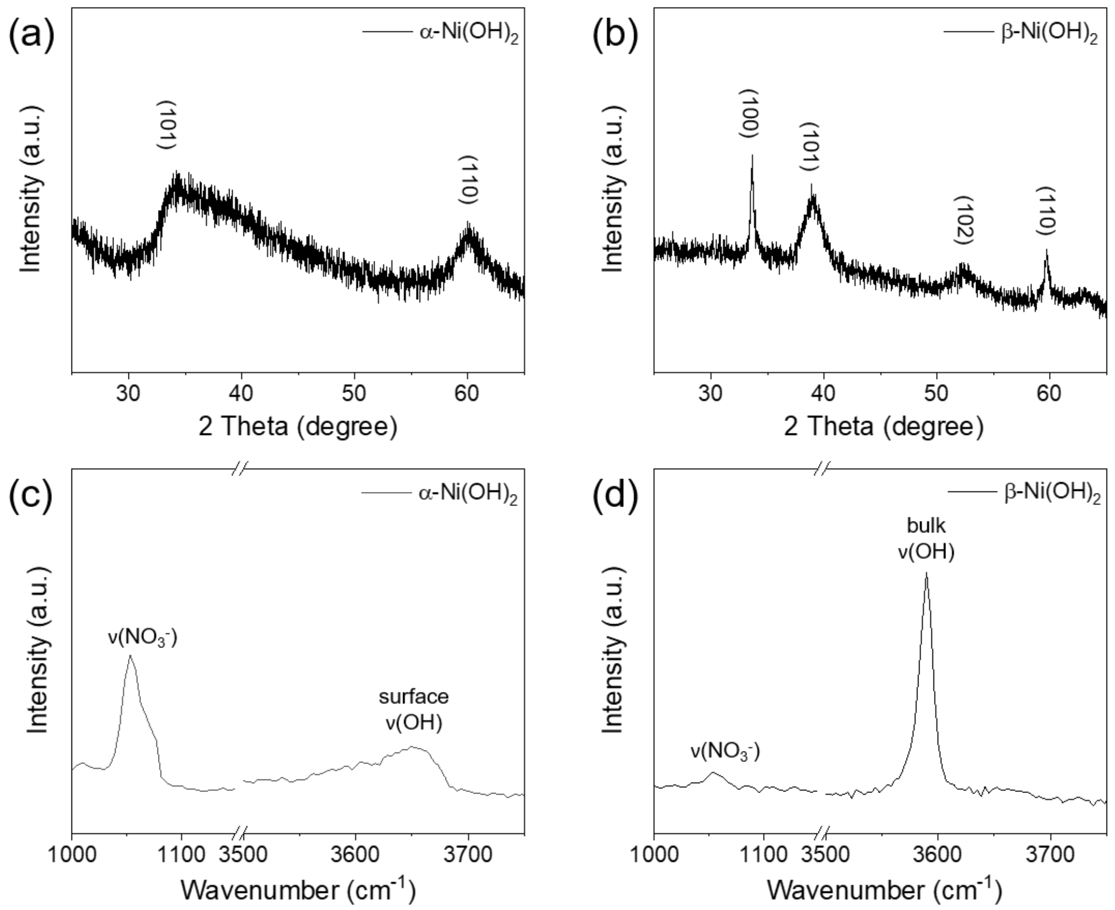

Figure S2. XRD patterns of the synthesized (a) α-$Ni(OH)_2$ and (b) β-$Ni(OH)_2$. Raman spectra of the synthesized (c) α-$Ni(OH)_2$ and (d) β-$Ni(OH)_2$.

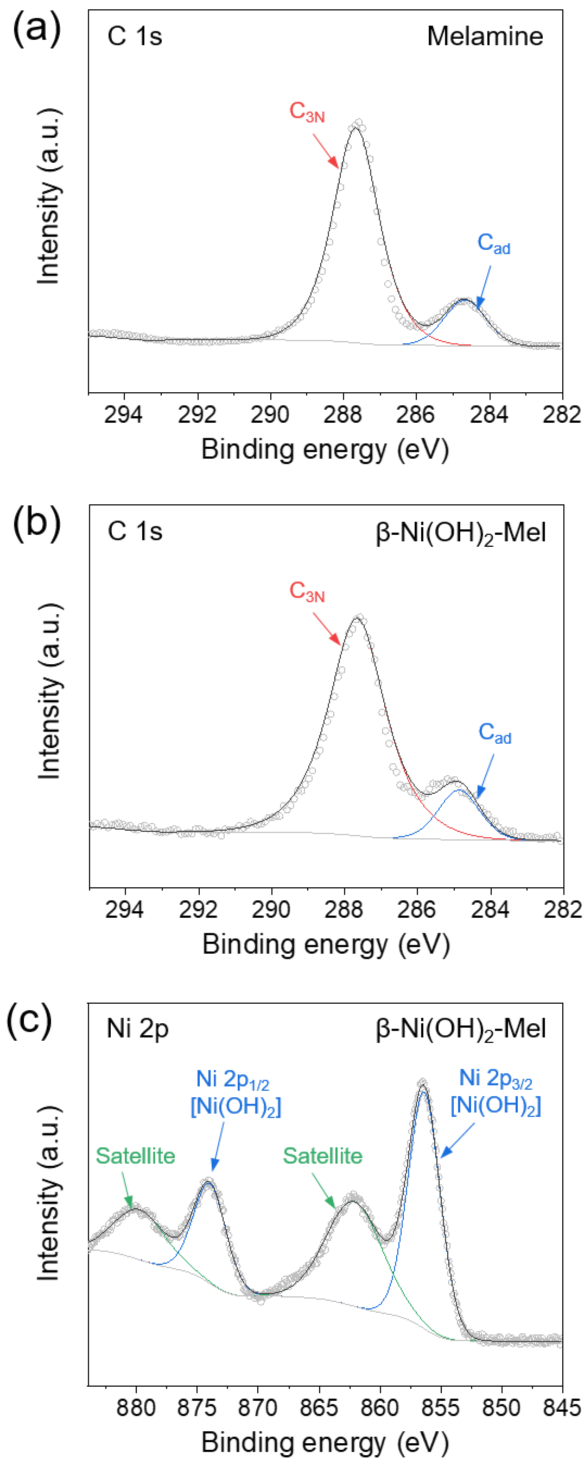

Figure S3. XPS C 1s spectra of (a) melamine and (b) β-$Ni(OH)_2$-Mel. (c) XPS Ni 2p spectrum of β-$Ni(OH)_2$-Mel.

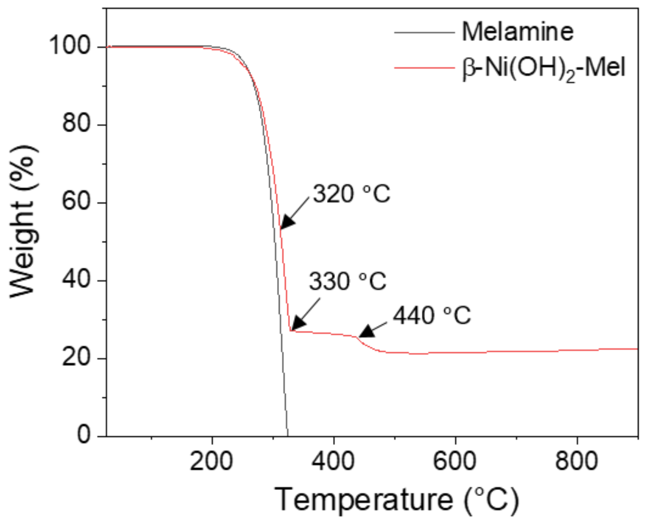

Figure S4. TGA curves of melamine and β-$Ni(OH)_2$-Mel under a nitrogen atmosphere.

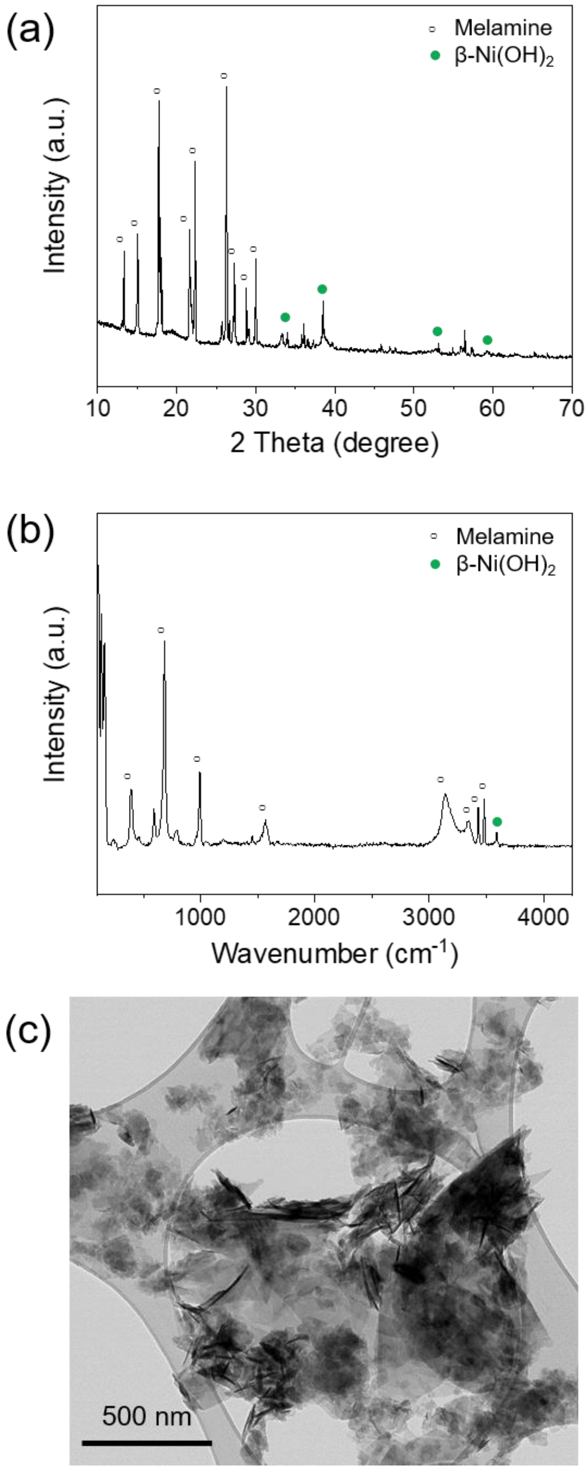

Figure S5. (a) XRD pattern, (b) Raman spectrum, and (c) TEM image of β-$Ni(OH)_2$-Mel_320.

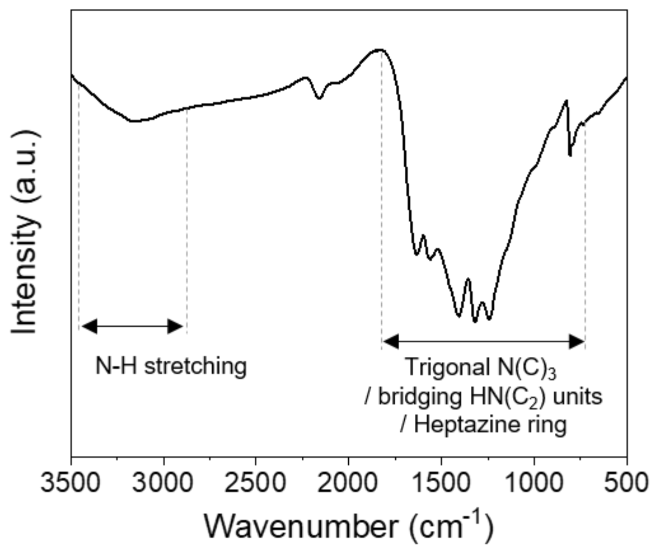

Figure S6. FT-IR spectrum of β-$Ni(OH)_2$-Mel_600.

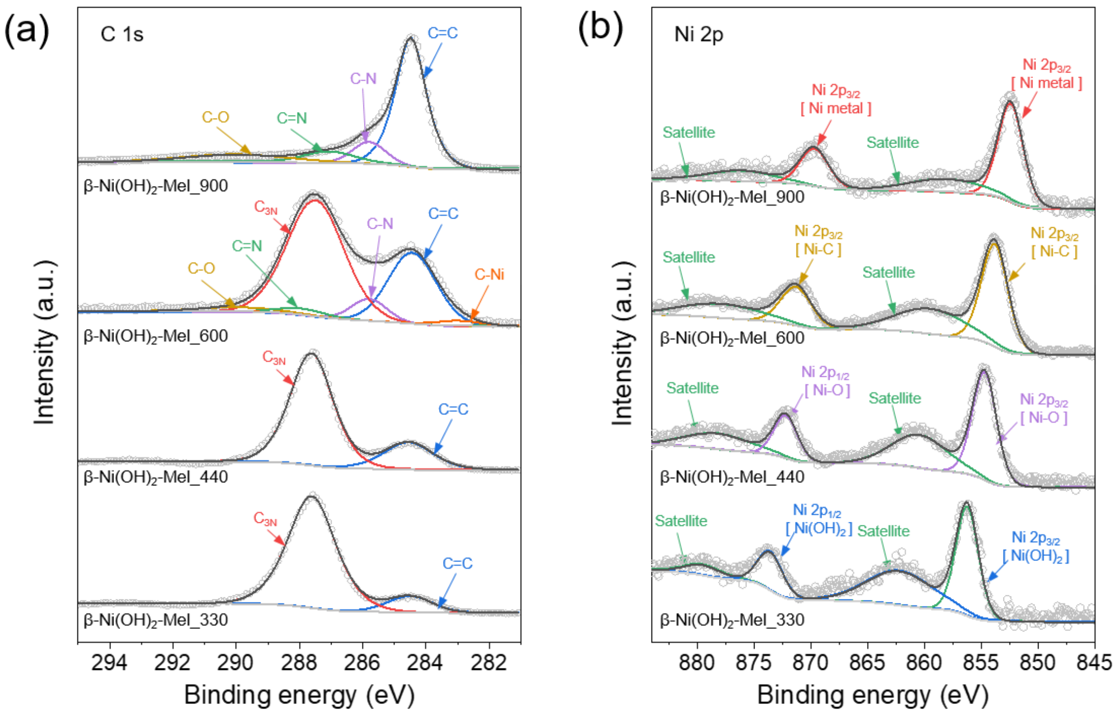

Figure S7. XPS (a) C 1s spectra and (b) Ni 2p spectra of β-$Ni(OH)_2$-Mel_330, 440, 600, and 900.

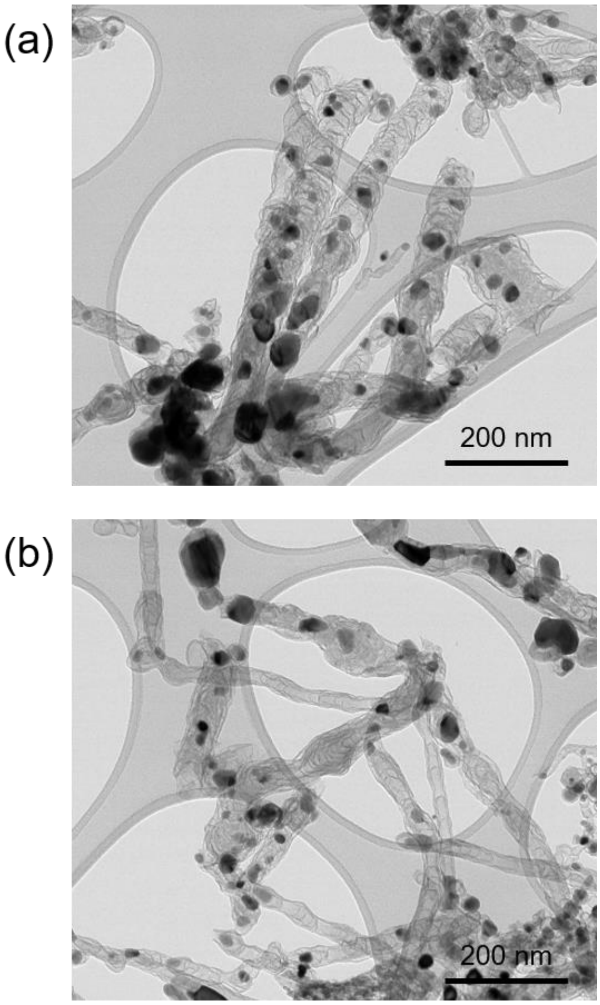

Figure S8. (a, b) TEM images of β-$Ni(OH)_2$-Mel_800.

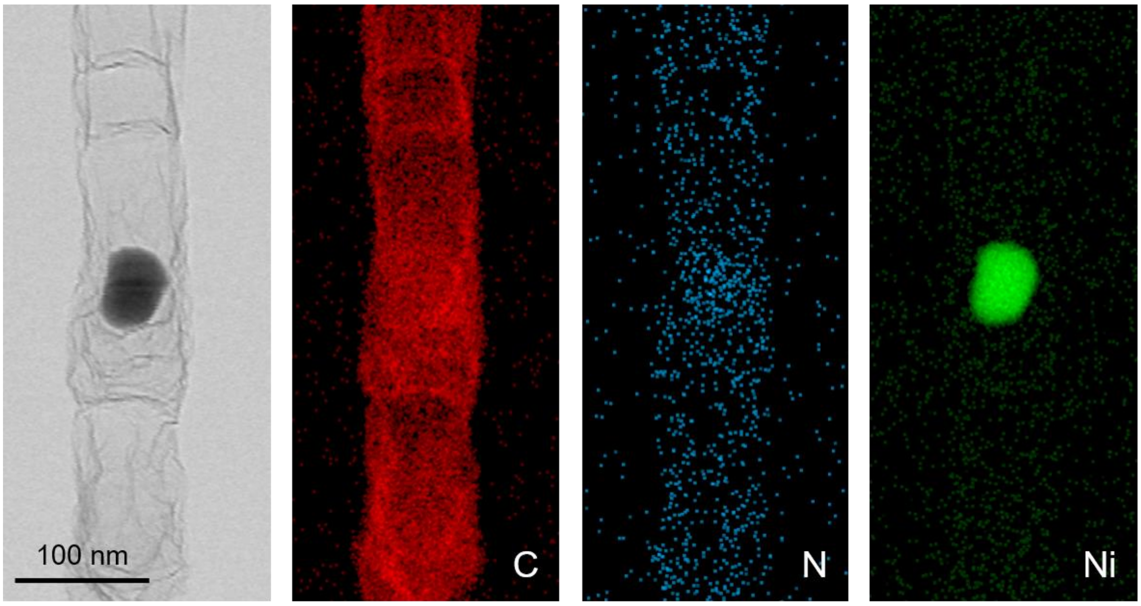

Figure S9. TEM image of Ni@N-IGN and elemental maps of carbon, nitrogen, and nickel.

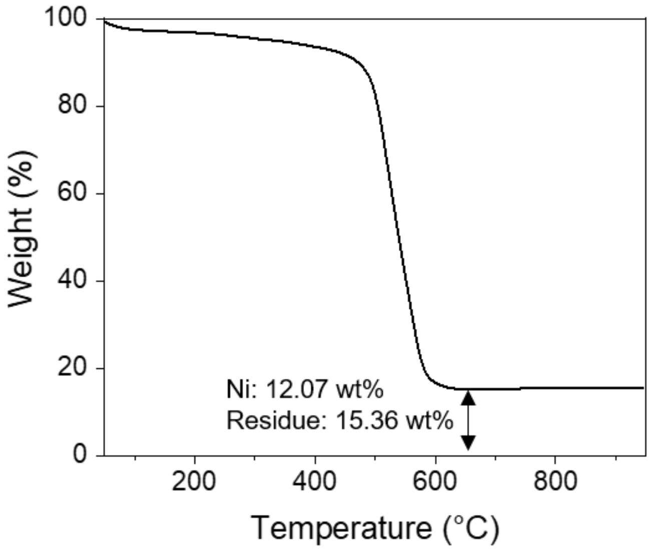

Figure S10. TGA curve of Ni@N-IGN under an air atmosphere.

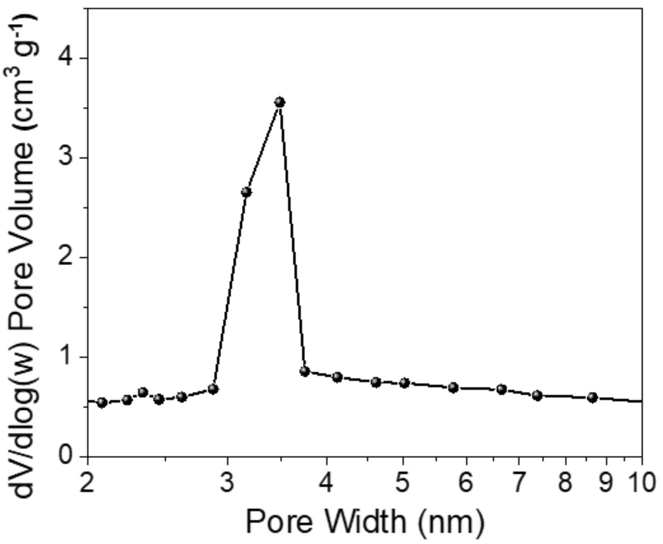

Figure S11. Pore size distribution of Ni@N-IGN.

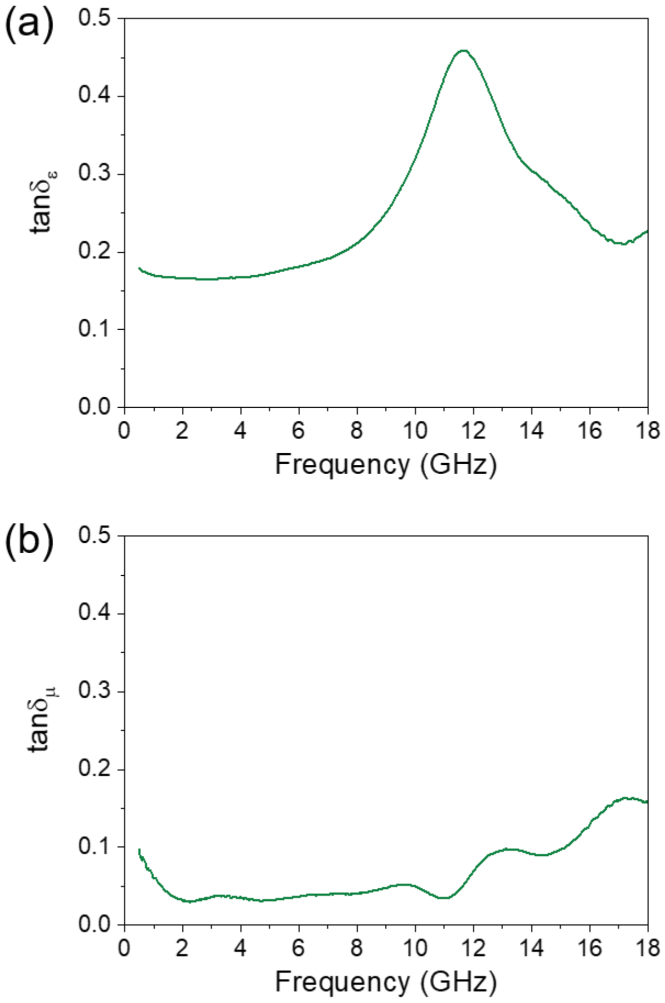

Figure S12. (a) Dielectric loss tangent and (b) magnetic loss tangent of the Ni@N-IGN composite.

Table S1. Summary of recent studies on electromagnetic absorption properties of carbon/magnetic metal hybrid materials.

Carbon	Metal	Ratio	RL (dB)	GHz	t (mm)	Ref.
N-doped graphitic nanocube	Ni	5	-62.1	12.1	2.45	This work
2D carbon sheet	Fe/TiO_2	40	-51.8	6.6	3	[1]
Porous amorphous carbon	Fe/MnO@C	50	-45	-	5.5	[2]
Amorphous carbon	Fe	60	-59.7	6.0	3.5	[3]
MOF derived carbon	FeCoNi	38	-64.75	15.44	2.1	[4]
MOF derived carbon	CoNi	10	-61.02	13.68	2	[5]
Polydopamine derived carbon	Fe	60	-66.5	-	1.6	[6]
N-doped carbon / rGO	CoNi	30	-58.2	10.62	2.5	[7]
Mxene-CNT	Ni	30	-56.4	-	2.4	[8]
MWCNT	Co	12	-55	5.2	4	[9]
N-doped CNT	Ni@C	30	-41.5	5.2	1.7	[10]
NCNT/rGO	CoNi	25	-41.13	-	3.5	[11]
Edge oxidized graphene	FeCoNi	45.4	-68.0	8.4	2.33	[12]
N-doped rGO	$FeNi_3$	50	-57.2	16	1.45	[13]
rGO	$Co_{33}Ni_{67}$	50	-50	16	1.8	[14]
N-Doped Graphene	CoNi	35	-47.79	-	3.0	[15]
rGO	CoNi	60	-31	4.9	4.0	[16]
rGO	Fe	40	-36.5	5.64	3	[17]
Exfoliated graphene	$NiCo_2$	50	-30	11.7	1.6	[18]

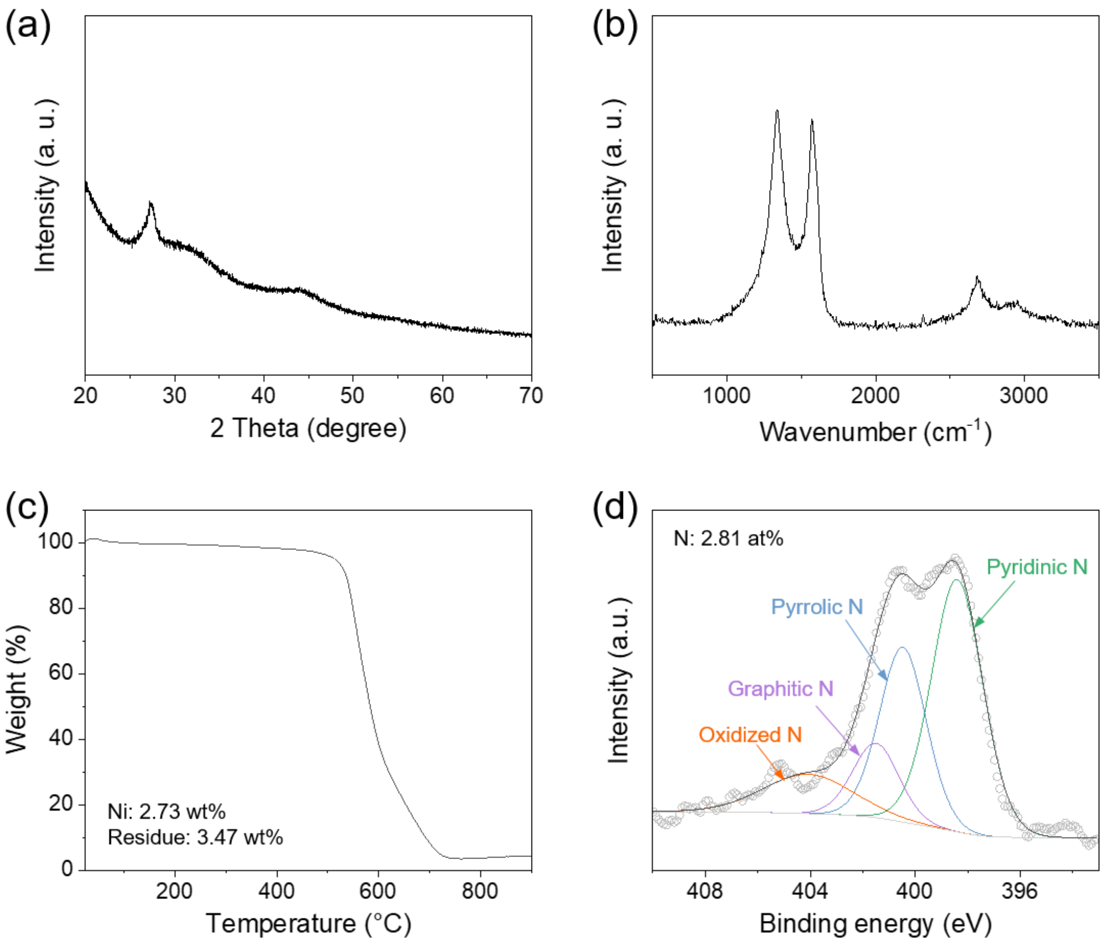

Figure S13. (a) XRD pattern, (b) Raman spectrum, (c) TGA curve, and (d) XPS N 1s spectrum of Ni@N-IGN after air oxidation and HCl etching.

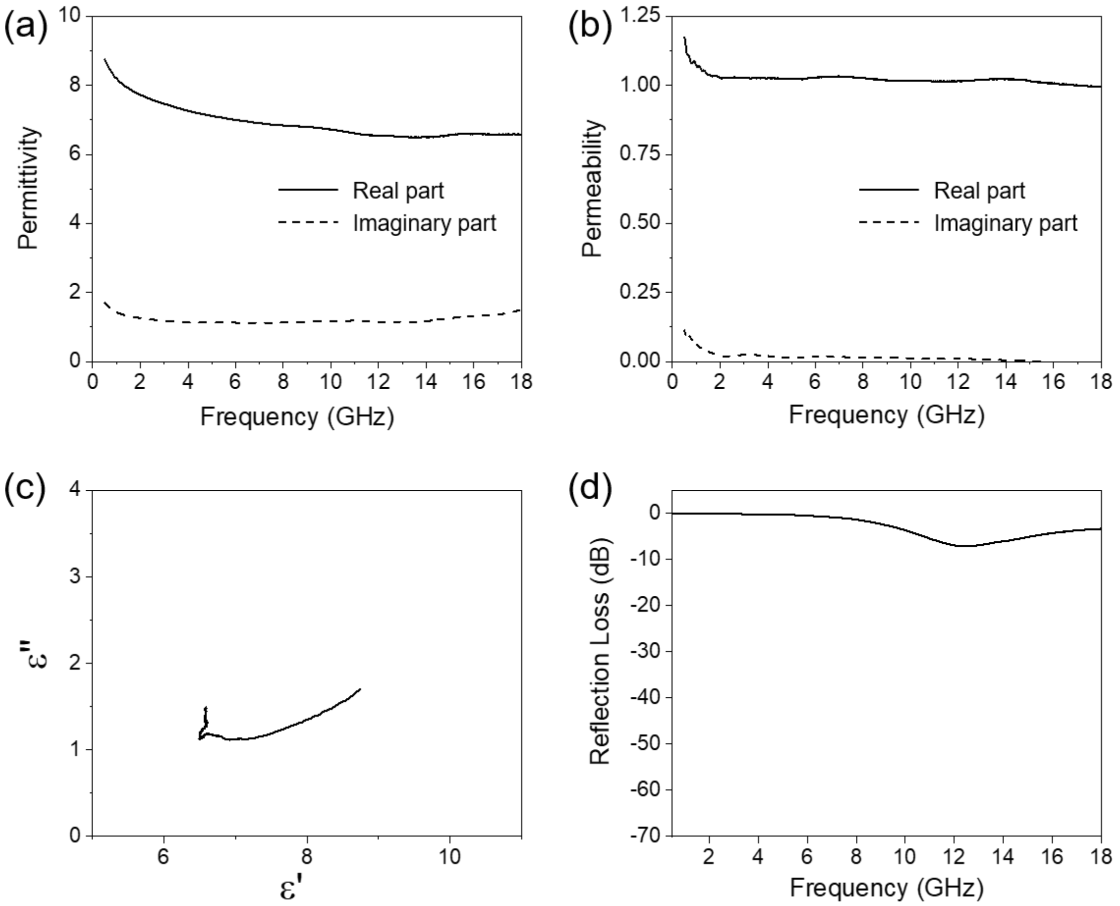

Figure S14. Real and imaginary (a) permittivity and (b) permeability of the a-Ni@N-IGN and paraffin composite. (c) Cole–Cole plot and (d) RL value of the a-Ni@N-IGN composite.

(a)

(b)

(c)

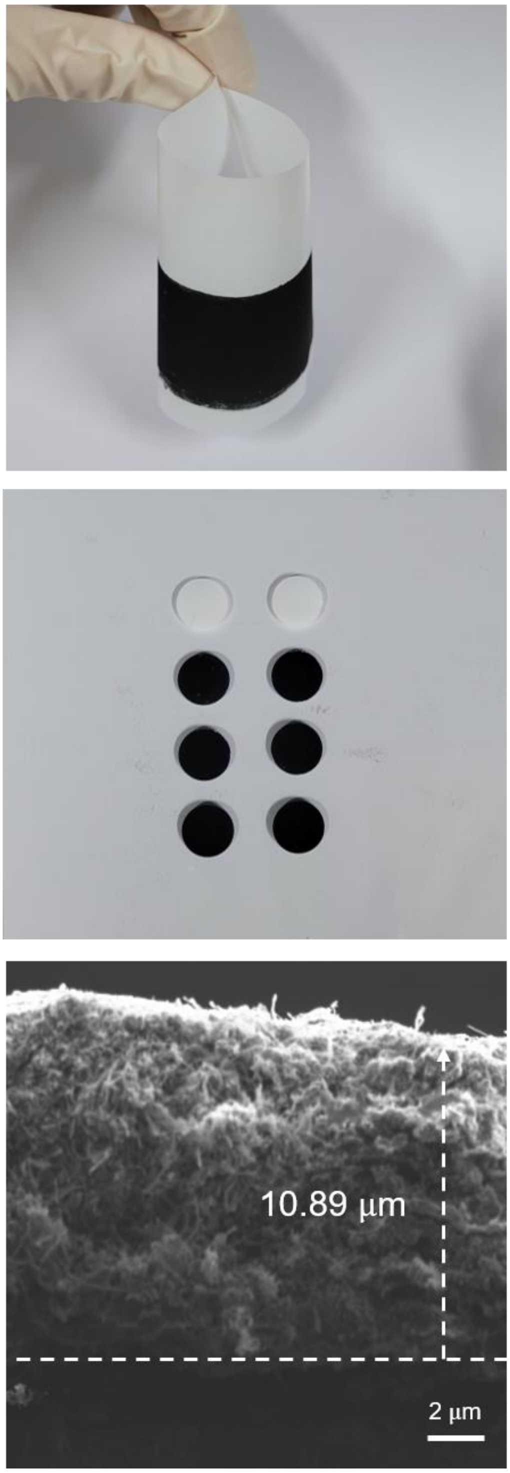

Figure S15. (a, b) Digital images and (c) SEM cross-sectional image of the Ni@N-IGN separator.

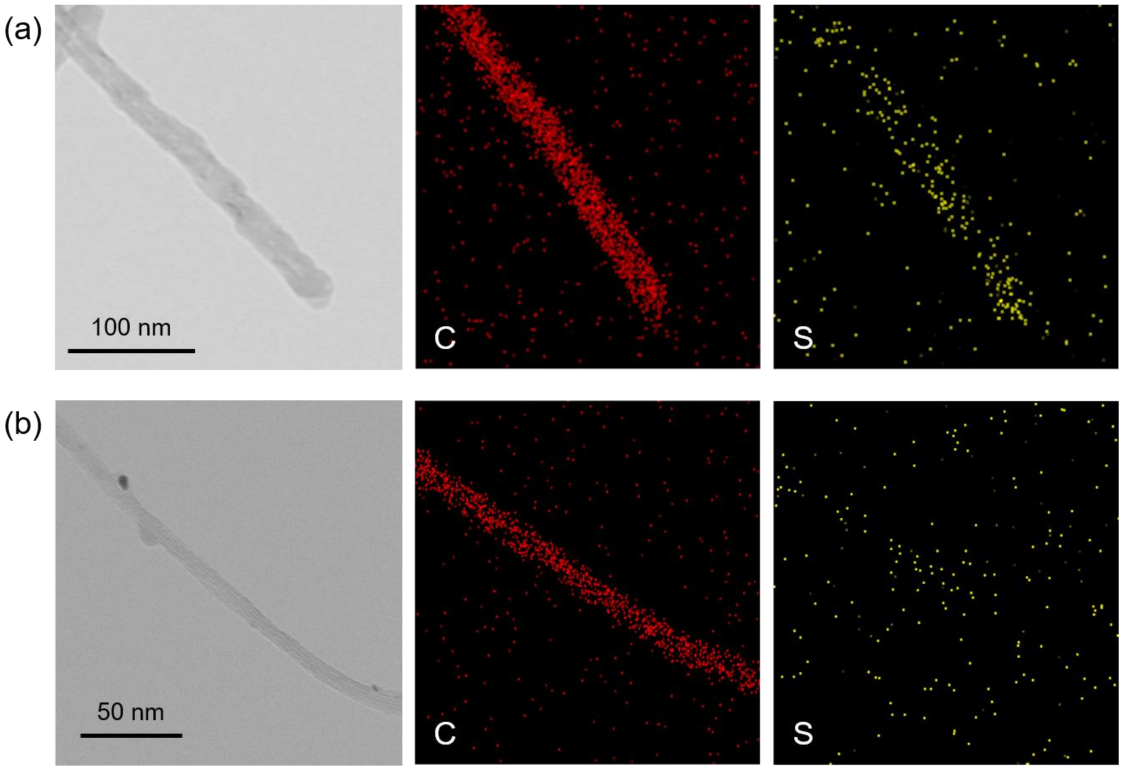

Figure S16. TEM images and elemental maps of carbon and sulfur for (a) aMWCNT and (b) SWCNT after the Li_2S_6 adsorption test.

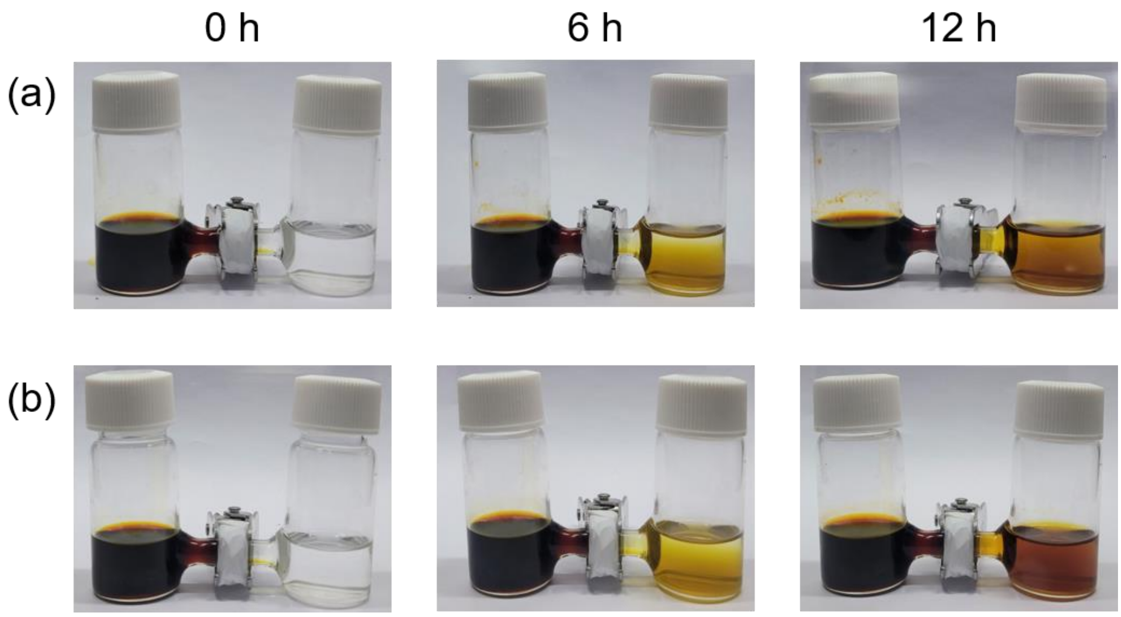

Figure S17. Visual inspection of the Li_2S_6 diffusion experiment for the (a) aMWCNT and (b) SWCNT separator.

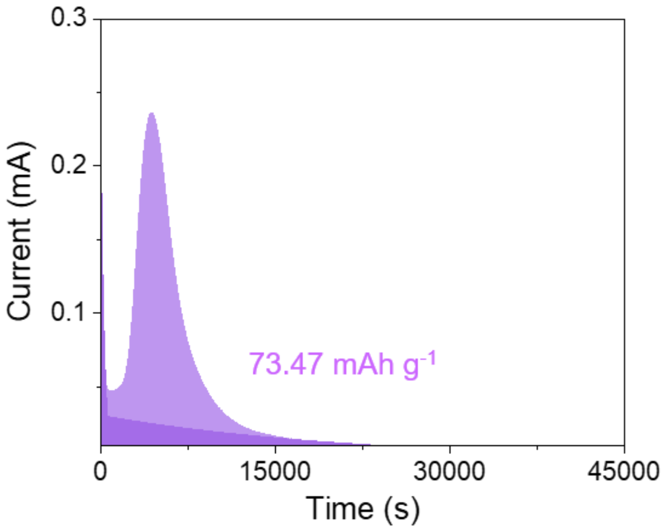

Figure S18. Potentiostatic discharge profile of the a-Ni@N-IGN cell.

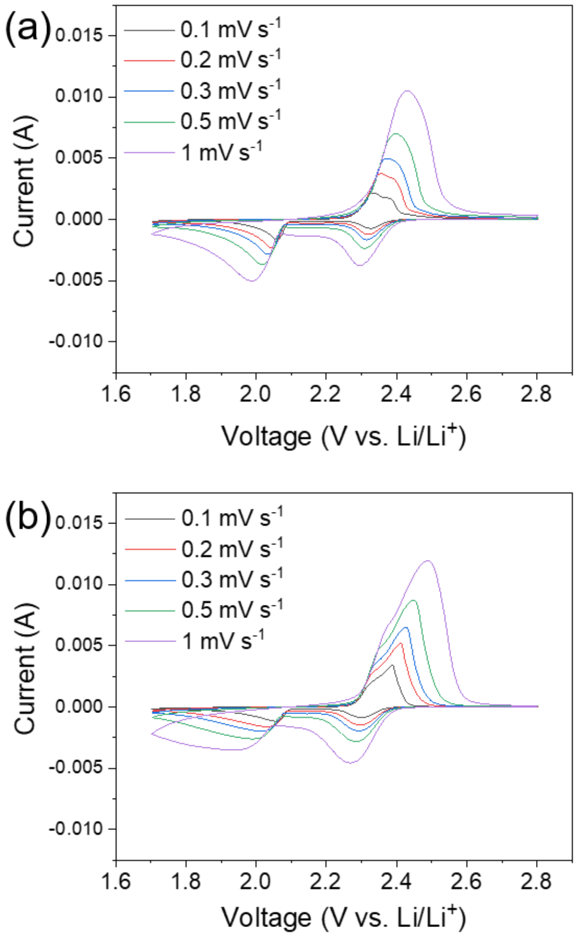

Figure S19. CV curves of Li-S cells with the (a) aMWNCT and (b) PP separator at various scan rates from 0.1 to 1 mV s^{-1}.

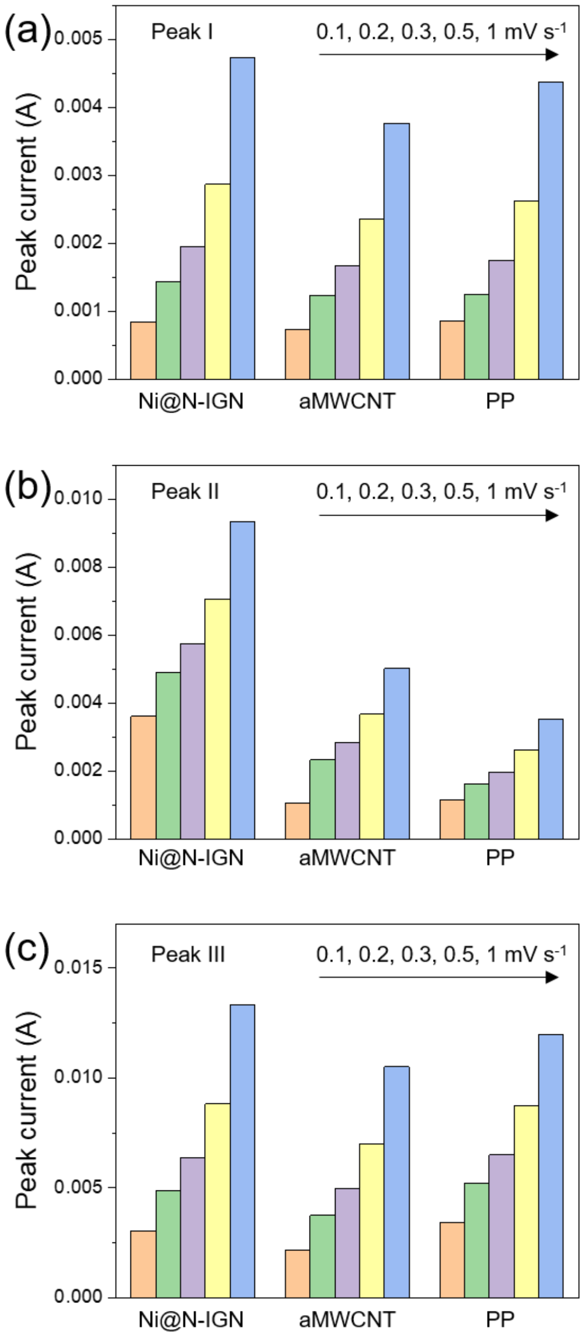

Figure S20. Peak currents of the three cells for (a) peak I, (b) peak II, and (c) peak III.

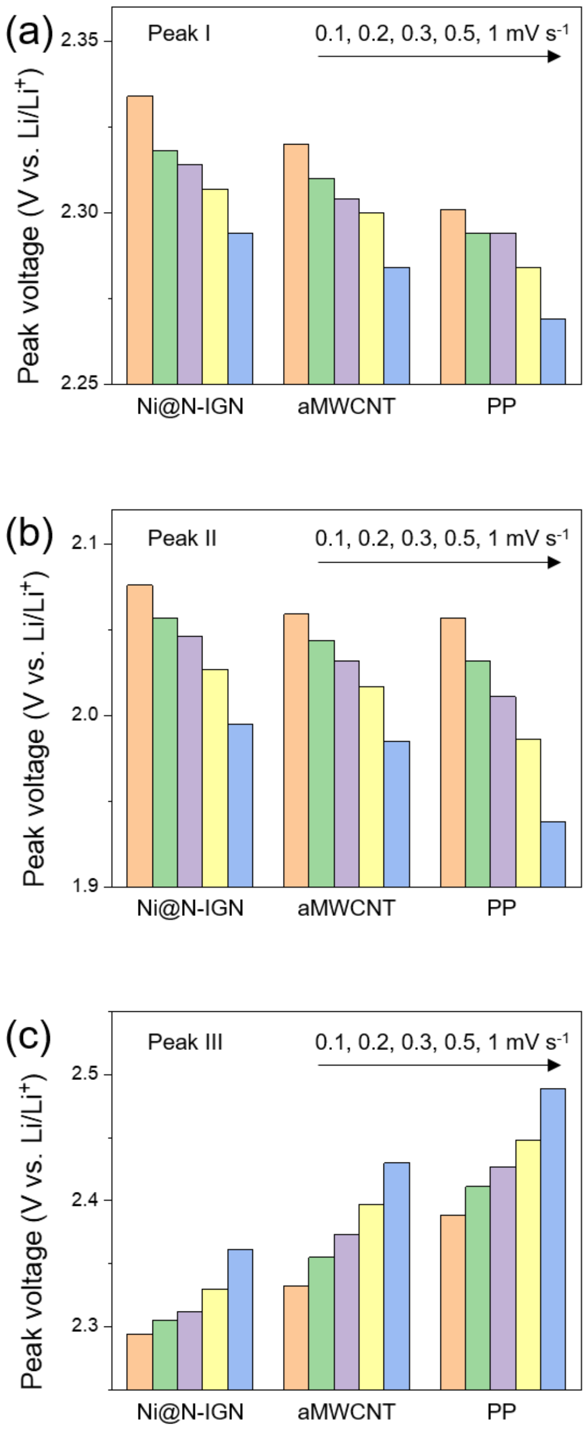

Figure S21. Peak potentials of three cells for (a) peak I, (b) peak II, and (c) peak III.

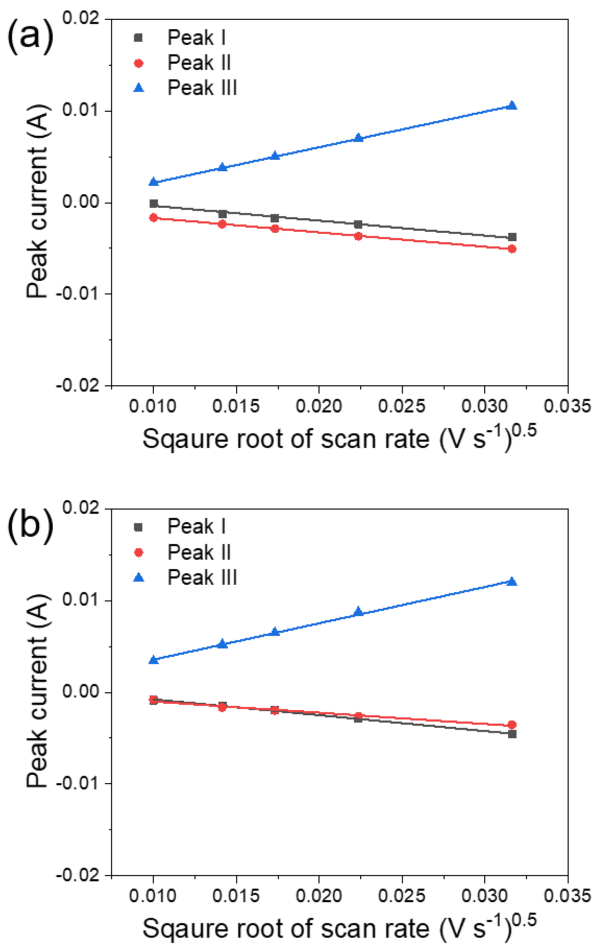

Figure S22. CV peak currents vs. the square root of the scan rates plot for the three characteristic peaks of the (a) aMWCNT and (b) PP separator cell.

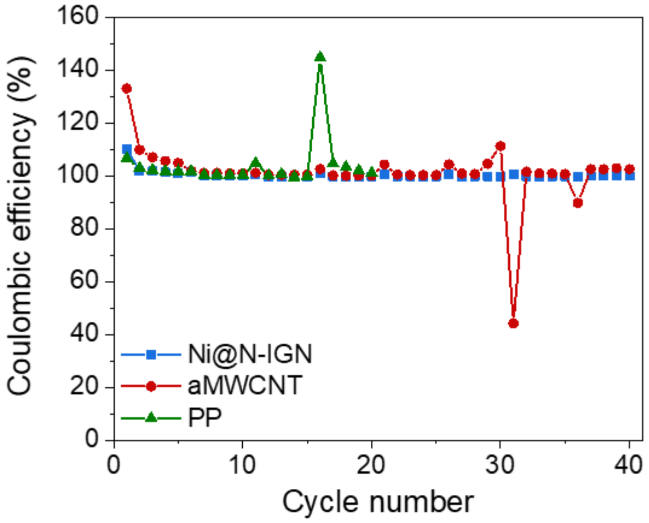

Figure S23. Coulombic efficiency of the three cells.

Table S2. Summary of average specific capacity (mAh g^{-1}) and Coulombic efficiency of three Ni@N-IGN separator cells at various C rates from 0.2 C to 5 C.

C-rate (C) / Cell #	0.2	0.5	1	2	3	4	5	Average Coulombic efficiency (%)
1	1323	1069	998	914	866	826	785	100.1
2	1354	1084	988	913	842	762	672	99.7
3	1308	1030	954	896	861	812	749	99.5
Average	1328	1061	980	908	856	800	735	-
Standard Deviation (%)	1.8	2.6	2.4	1.1	1.5	4.2	7.9	-

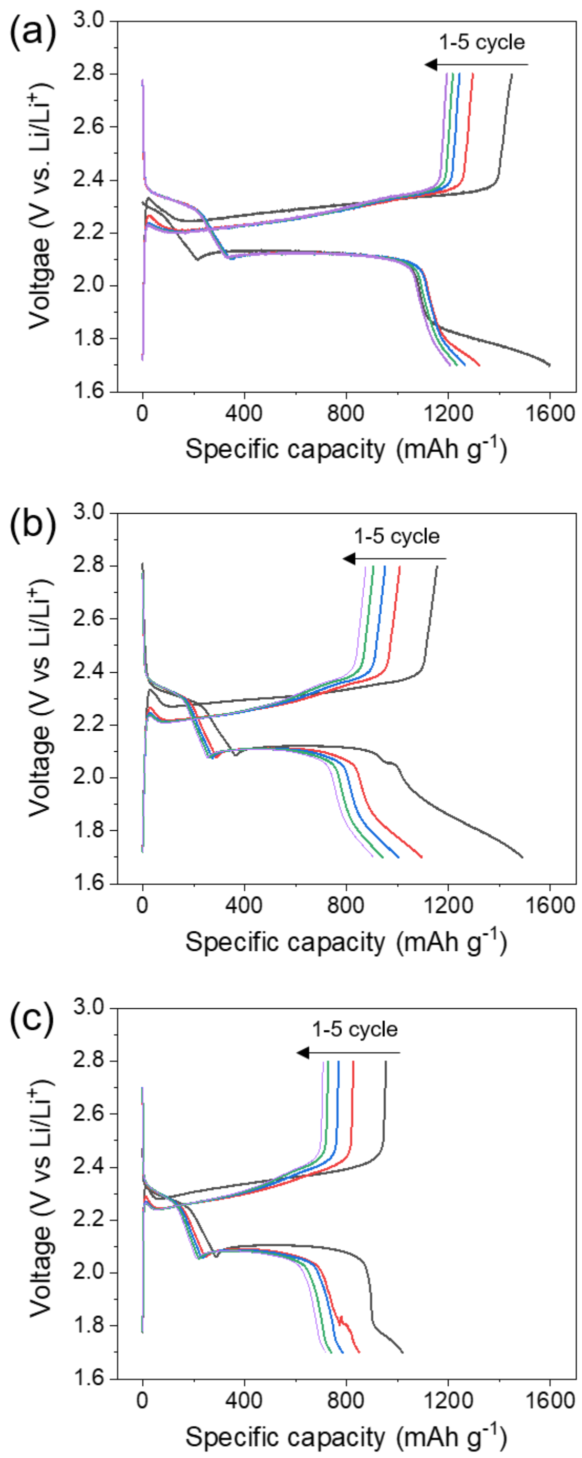

Figure S24. Charge/discharge voltage profiles of the (a) Ni@N-IGN, (b) aMWCNT, and (c) PP separator cell at 0.2 C for five cycles.

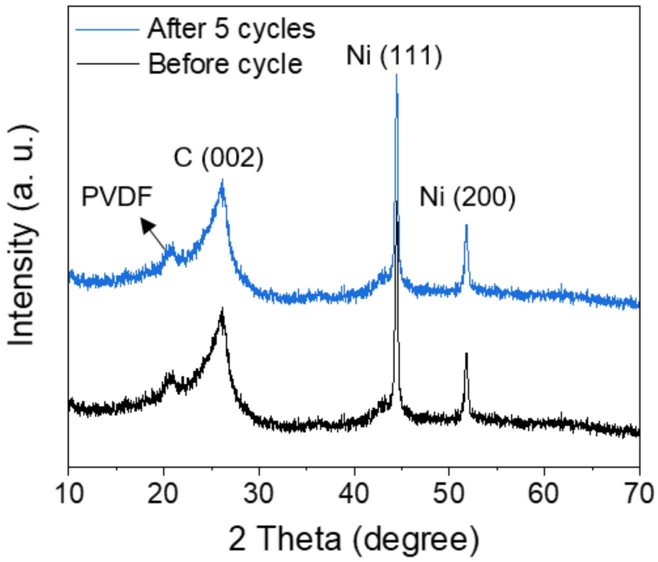

Figure S25. XRD patterns of the Ni@N-IGN separator before and after 5 cycles at 0.2 C.

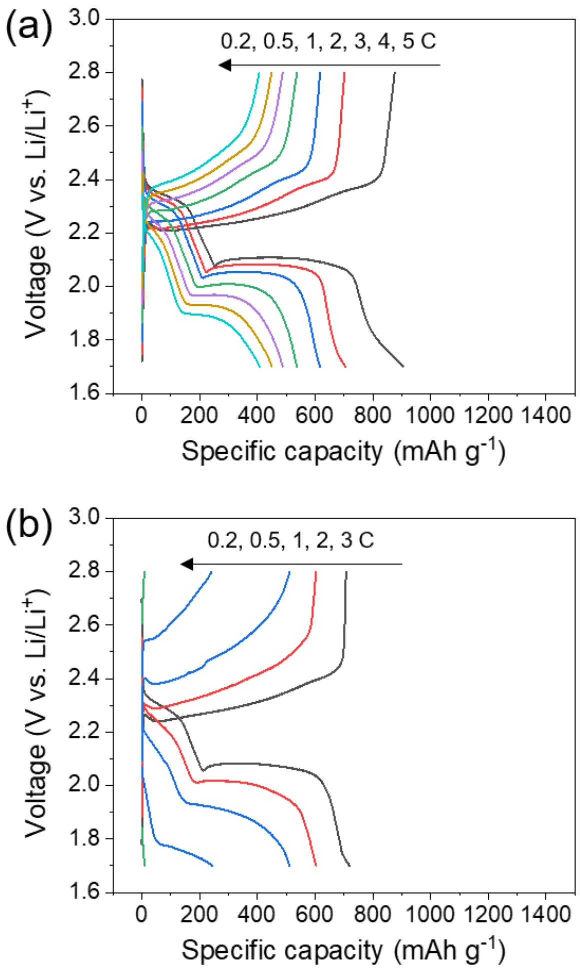

Figure S26. Charge/discharge voltage profiles of the (a) aMWCNT and (b) PP separator cell at various C rates from 0.2 to 5 C.

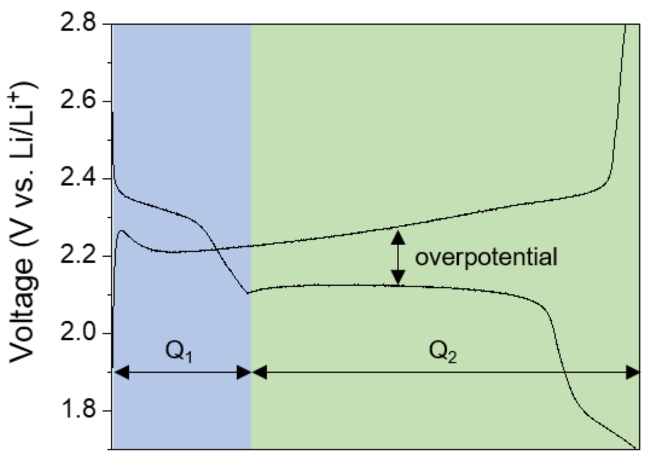

Figure S27. The charge/discharge voltage profile to distinguish capacity and measure overpotential.

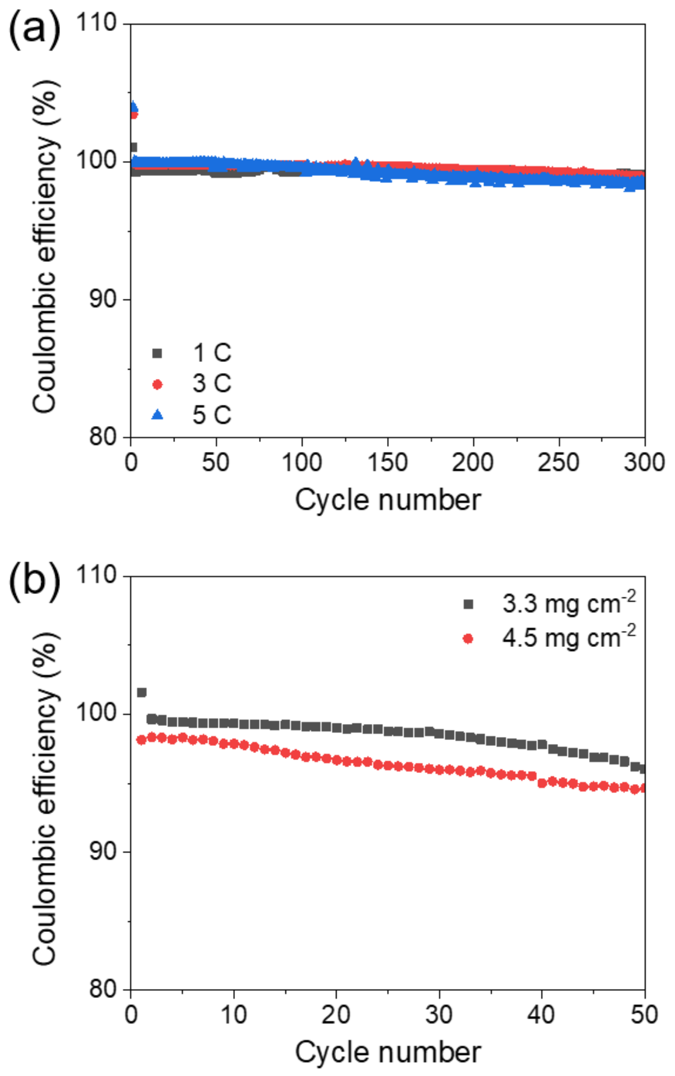

Figure S28. Coulombic efficiency of the Ni@N-IGN separator cell (a) at various C rates for extended cycles and (b) at 0.5 C with higher sulfur loading (3.3 and 4.5 mg cm^{-2}).

Table S3. Summary of specific capacity, capacity decay per cycle, and Coulombic efficiency of three Ni@N-IGN separator cells at 1, 3 and 5 C.

C-rate	Cell #	Initial capacity (mAh g^{-1})	300th capacity (mAh g^{-1})	Capacity decay per cycle (%)	Coulombic efficiency (%)
1 C	1	1067	874	0.06	99.3
	2	1006	729	0.09	99.6
	3	1081	869	0.07	99.7
	Average	1051	824	-	-
	Standard deviation (%)	3.8	10.0	-	-
3 C	1	850	714	0.05	99.6
	2	855	661	0.08	98.4
	3	857	678	0.07	99.0
	Average	854	684	-	-
	Standard deviation (%)	0.4	4.0	-	-
5 C	1	814	644	0.07	98.3
	2	809	632	0.07	99.4
	3	783	619	0.07	99.2
	Average	802	632	-	-
	Standard deviation (%)	2.1	2.0	-	-

Table S4. Summary of specific capacity, capacity decay per cycle, and Coulombic efficiency of three Ni@N-IGN separator cell with higher sulfur loading conditions (3.3 and 4.5 mg cm^{-2}) at 0.5 C.

Sulfur loading (mg cm^{-2})	Initial capacity (mAh g^{-1})	50th capacity (mAh g^{-1})	Capacity decay per cycle (%)	Coulombic efficiency (%)
3.3	1043	1005	0.07	98.5
3.3	1045	1001	0.08	96.8
3.2	1071	986	0.16	97.9
Average	1053	997	-	-
Standard deviation (%)	1.5	1.0	-	-
4.5	737	695	0.11	96.4
4.5	842	668	0.41	96.1
4.6	816	652	0.40	96.8
Average	798	672	-	-
Standard deviation (%)	6.9	3.2	-	-

References

[1] B. Deng, Z. Xiang, J. Xiong, Z. Liu, L. Yu, W. Lu, *Nanomicro Lett.* **2020**, *12*, 55.

[2] G. He, Y. Duan, H. Pang, *Nanomicro Lett.* **2020**, *12*, 57.

[3] X. Li, W. Dong, C. Zhang, W. Guo, C. Wang, Y. Li, H. Wang, *Compos. Part A Appl. Sci. Manuf.* **2021**, *140*, 106202.

[4] J. Ouyang, Z. He, Y. Zhang, H. Yang, Q. Zhao, *ACS Appl. Mater. Interfaces* **2019**, *11*, 39304.

[5] Y.-L. Wang, S.-H. Yang, H.-Y. Wang, G.-S. Wang, X.-B. Sun, P.-G. Yin, *Carbon* **2020**, *167*, 485.

[6] S. Gao, Y. Zhang, H. Xing, H. Li, *Chem. Eng. J.* **2020**, *387*, 124149.

[7] Y. Zhao, W. Wang, J. Wang, J. Zhai, X. Lei, W. Zhao, J. Li, H. Yang, J. Tian, J. Yan, *Carbon* **2021**, *173*, 1059.

[8] X. Li, W. You, C. Xu, L. Wang, L. Yang, Y. Li, R. Che, *Nanomicro Lett.* **2021**, *13*, 157.

[9] M. A. Kazakova, N. V. Semikolenova, E. Y. Korovin, V. A. Zhuravlev, A. G. Selyutin, D. A. Velikanov, S. I. Moseenkov, A. S. Andreev, O. B. Lapina, V. I. Suslyaev, M. A. Matsko, V. A. Zakharov, J.-B. d. E. d. Lacaillerie, *Compos. Sci. Technol.* **2021**, *207*, 108731.

[10] D. Liu, Y. Du, P. Xu, F. Wang, Y. Wang, L. Cui, H. Zhao, X. Han, *J. Mater. Chem. A* **2021**, *9*, 5086.

[11] X. Zhang, X. Zhang, H. Yuan, K. Li, Q. Ouyang, C. Zhu, S. Zhang, Y. Chen, *Chem. Eng. J.* **2020**, *383*, 123208.

[12] T. Kim, J. Lee, K. Lee, B. Park, B. M. Jung, S. B. Lee, *Chem. Eng. J.* **2019**, *361*, 1182.

[13] J. Feng, Y. Zong, Y. Sun, Y. Zhang, X. Yang, G. Long, Y. Wang, X. Li, X. Zheng, *Chem. Eng. J.* **2018**, *345*, 441.

[14] H. Pan, M. Xu, Q. Qi, X. Liu, *RSC Adv.* **2017**, *7*, 43831.

[15] X. Zhang, F. Yan, S. Zhang, H. Yuan, C. Zhu, X. Zhang, Y. Chen, *ACS Appl. Mater. Interfaces* **2018**, *10*, 24920.

[16] G. Sun, H. Wu, Q. Liao, Y. Zhang, *Nano Res.* **2018**, *11*, 2689.

[17] Y. Li, M. Yu, P. Yang, J. Fu, *Ind. Eng. Chem. Res.* **2017**, *56*, 8872.

[18] R. Yang, B. Wang, J. Xiang, C. Mu, C. Zhang, F. Wen, C. Wang, C. Su, Z. Liu, *ACS Appl. Mater. Interfaces* **2017**, *9*, 12673.
